# Mechanism, Material, Design, and Implementation Principle of Two-Dimensional Material Photodetectors

**DOI:** 10.3390/nano11102688

**Published:** 2021-10-12

**Authors:** Cheng Yang, Guangcan Wang, Maomao Liu, Fei Yao, Huamin Li

**Affiliations:** 1 School of Physics and Electronics, Shandong Normal University, Jinan 250014, China; wangguangcan@126.com; 2 Department of Electrical Engineering, University at Buffalo, The State University of New York, Buffalo, NY 14260, USA; maomaoli@buffalo.edu; 3 Department of Materials Design and Innovation, University at Buffalo, The State University of New York, Buffalo, NY 14260, USA; feiyao@buffalo.edu

**Keywords:** 2D materials, photodetectors, photocurrent-enhanced structure

## Abstract

Two-dimensional (2D) materials may play an important role in future photodetectors due to their natural atom-thin body thickness, unique quantum confinement, and excellent electronic and photoelectric properties. Semimetallic graphene, semiconductor black phosphorus, and transition metal dichalcogenides possess flexible and adjustable bandgaps, which correspond to a wide interaction spectrum ranging from ultraviolet to terahertz. Nevertheless, their absorbance is relatively low, and it is difficult for a single material to cover a wide spectrum. Therefore, the combination of phototransistors based on 2D hybrid structures with other material platforms, such as quantum dots, organic materials, or plasma nanostructures, exhibit ultra-sensitive and broadband optical detection capabilities that cannot be ascribed to the individual constituents of the assembly. This article provides a comprehensive and systematic review of the recent research progress of 2D material photodetectors. First, the fundamental detection mechanism and key metrics of the 2D material photodetectors are introduced. Then, the latest developments in 2D material photodetectors are reviewed based on the strategies of photocurrent enhancement. Finally, a design and implementation principle for high-performance 2D material photodetectors is provided, together with the current challenges and future outlooks.

## 1. Introduction

Photodetectors are one of the key components in modern multifunctional technologies that can convert light signals into electrical signals [1,2]. High-performance photodetectors play an important role in many areas of daily life, including imaging [3], environmental monitoring [4], optical communications [5], and military and security inspections [6]. Photodetectors based on 2D materials and their heterostructures have recently attracted considerable interest [7]. First, the atoms of 2D materials can be arranged by strong in-plane covalent bonds or ionic bonds to form a planar structure [8]. Weak van der Waals (vdW) interactions along the out-of-plane direction can stack these thin layers to form heterostructures [9,10]. Even the monolayer or single-layer 2D materials with a sub-nanometer thickness still interact strongly with light [11,12]. Second, the surface of the 2D material is naturally passivated [13]. There are no dangling bonds on the surface [13], so the vdW interaction allows different materials to be overlayed under lattice mismatch constraints [9]. Third, 2D materials, such as graphene [14,15], black phosphorous (BP) [16], and transition metal dichalcogenides [17], can achieve a broad response range in the entire electromagnetic spectrum due to their various bandgaps. Fourth, some 2D materials (such as graphene and BP) have shown the potential to serve as atom-thin information transmission channels [18], because their optoelectronic properties can be easily adjusted by local fields (such as charged trap states [19], the electric field, the ferroelectric field [20,21], and the magnetic field) [22,23]. Compared to conventional thin-film materials, 2D materials are considered competitive and promising material candidates for high-performance photodetector applications [24].

On the other hand, 2D materials and their photodetectors also face challenges and issues [25]. The ultra-high mobility of graphene makes it suitable for high-speed photodetectors. Nevertheless, its single-atom-thin body thickness and zero bandgaps limit its light absorption [26,27,28], external quantum efficiency [29], and responsivity [18]. The graphene photodetectors suffer from relatively large dark currents, which are usually related to the lack of a bandgap [30]. Various methods have been proposed to introduce band gaps in graphene, such as scaling graphene down to nanoribbons or applying a strain effect [31]; However, as the bandgap opens, the broadband response of the graphene photodetectors are compromised. The 2D transition metal dichalcogenides (TMDs) with larger bandgaps can overcome the shortcomings of graphene [32,33,34]. Whereas the light response wavelength range is limited from ultraviolet to near-infrared due to their optical bandgaps. Moreover, the response speed of the TMD photodetectors is relatively slow because of the low mobilities and the trapping effect for photo charge carriers [35]. BP has an intermediate bandgap between graphene and TMDs, covering a broad light response spectrum from near-infrared to mid-infrared. However, its instability in the air is a major challenge for practical applications. Overall, low-dark current and high-responsivity photodetectors are needed for technological development [36,37,38]. If these challenges can be solved, the 2D material photodetectors are expected to achieve many unparalleled achievements, including ultra-high responsivity (10^10^ A/W) [39], ultra-fast light response (0.4 ps) [40], an ultra-wide detection band (10.6 μm) [41], and ultra-sensitive photodetection (10^16^ Jones) [42].

This paper first briefly describes the underlying mechanism of 2D photodetectors and the key metrics for performance evaluation. Subsequently, we summarize photocurrent enhancement methods for 2D material photodetectors and present the recent advances in 2D material photodetectors. Finally, we discuss the strategies to overcome future challenges and implement 2D material photodetectors with enhanced and balanced performance.

## 2. Photocurrent Generation Mechanism

Photodetectors utilize different light-induced effects through electrical measurements, classified according to the nature of the physical effects caused by the incident radiation: photonic detectors rely on electron–hole pairs produced directly by light excitation, while thermal detectors rely on the thermalization of hot carriers by changes in electron or lattice temperature. In the thermal-type detectors, the absorbed incident radiation changes the temperature of the material, which in turn leads to a measurable change in the physical quantity of electrical detection. Generally, the thermal detectors are not sensitive to wavelength, are slower, and are usually cheaper than the photon detectors. On the contrary, in the photonic detector, the electrical output signal directly comes from the charged photocarriers generated during the photoexcitation process, thereby improving the signal-to-noise ratio and a providing a fast response. Here we focus on the discussion of the photonic detectors because of their overall better performance compared to the thermal detectors. However, semimetals are promising candidates to achieve highly sensitive, low-energy photodetection with ultra-fast operation. By exploiting the shift current response of the Weyl semimetals, the Berry field near the Weyl nodes is enhanced, and the responsivity will be improved by at least two orders of magnitude.

### 2.1. Photonic-Type Mechanism

#### 2.1.1. Photovoltaic (PV) Effect

**Figure 1:**
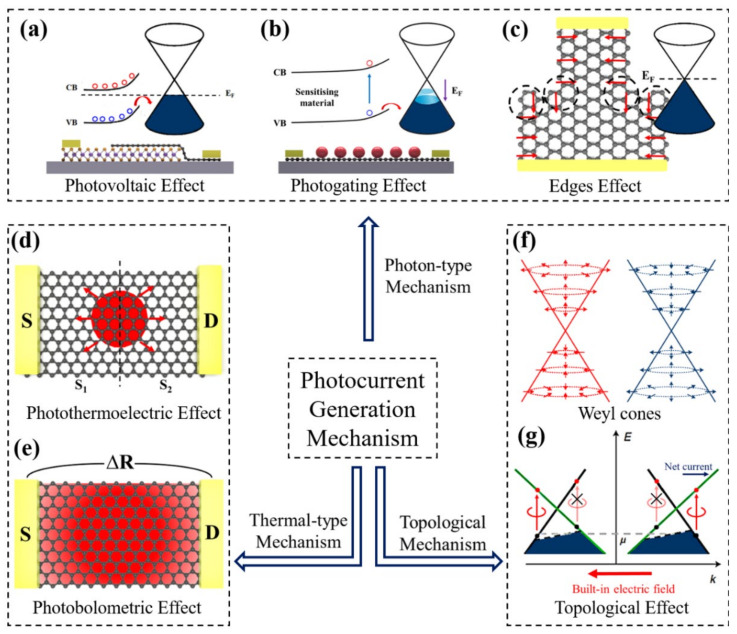
Photodetection mechanisms. (**a**) Photovoltaic effect. (**b**) Photogating effect. (**c**) Graphene Edges Effect. (**d**) Photothermoelectric effect. (**e**) Photobolometric Effect. (**f**) Sketch of a pair of Weyl cones. (**g**) Schematics of the chiral selection rule and circular photogalvanic effect response from a pair of Weyl cones in momentum space. The grey dashed line denotes the Fermi level μ without applying a built-in electric field. Black crosses mark the forbidden transitions.

**Figure 2:**
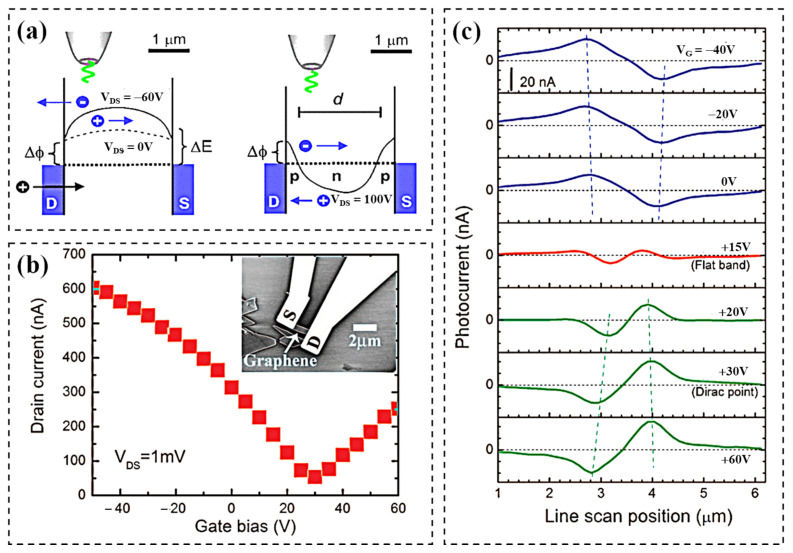
Photovoltaic Effect. (**a**) Band diagrams at VGS = 0 V (**dashed line**) and VGS = −60 V (**solid line**) were obtained by numerical integration of the photocurrent profiles. Δϕ describes the pinning of the Fermi level. Arrows indicate the flow of electrons and holes. (**b**) Electrical transport characteristic of a graphene transistor (drain current vs gate bias) at a drain bias of 1 mV. Inset: Scanning electron micrograph of the graphene transistor. (**c**) Photocurrent line scan profiles under different biases. (**a**) Reproduced with permission from [44]. Copyright American Physical Society, 2009. (**b**) and (**c**) reproduced with permission from [45]. Copyright 2009 American Chemical Society, 2009.

We start with the PV effect (Figure 1a) to explain the photoresponse in field-effect transistors. Many studies have demonstrated that photocurrents in field-effect transistors are generated in differently doped regions or near p–n junctions of 2D materials [43,44,45]. Electron–hole pairs are generated under light, and the separation of electrons and holes leads to the formation of photocurrent under the electric field. The built-in electric field drives the electron–hole pair separation to a limited extent, while an externally applied voltage can assist (Figure 2a). In the case of a weak built-in electric field, the photogenerated carriers recombine rapidly, so there is no contribution to the external photocurrent (Figure 2b,c). Therefore, a higher photocurrent can be obtained by rationally using a surface chemical modification of 2D materials to expand their bandgaps, and further construct devices with asymmetric source drain by forming a strong built-in electric field in contact with the metal. The PV effect usually exerts its advantages in optoelectronic devices based on the p–n junctions [46]. As the built-in potential of the p–n junction generates an electric field, the p–n junction can also extend the life of charge carriers. In addition, the van der Waals structure also provides spatial uniformity and a clean interface with low trap states. Shin et al. demonstrated a high-sensitivity photodetector based on WSe_2_ and MoS_2_ with a van der Waals heterostructure [47]. The photocurrent was effectively enhanced by shortening the transmission distance in the heterojunction. It was confirmed that the p–n junction plays a good role in separating carriers in the device. The photodetector has good performance, including a light responsivity of 2700 A/W, detectivity of 5 × 10^11^ Jones, and a response time of 17 ms. However, the PV effect was not the only mechanism found in the differently doped regions of 2D materials or near the graphene p–n junction. Usually, the hot carriers generated by 2D materials under the light will cause significant photothermoelectric (PTE) effects and photobolometric effects. In the report by Xu et al., without excluding the built-in electric field, the consistency of theoretical explanation and experimental results strongly indicate that the PTE effect may also be the origin of photocurrent in p–n junction devices [48].

#### 2.1.2. Photogating (PG) Effect

In the photoconductive (PC) effect, the photo-induced additional carriers lead to an increase in the concentration of free carriers, which leads to a decrease in the resistance of the semiconductor. The excess carriers are separated by the applied bias voltage, resulting in a photocurrent. In dark conditions, the finite carriers are driven by the applied bias voltage that can produce a small dark current. Under illumination, the absorbed photons produce electron–hole pairs separated and driven by the applied bias voltage, resulting in a current more significant than the dark current. The photogenerated excess carrier density increases the conductance. If the bias voltage is not applied, the photocurrent of the photoconductive effect cannot be generated, which is entirely different from the bias current of the PV effect.

**Figure 3:**
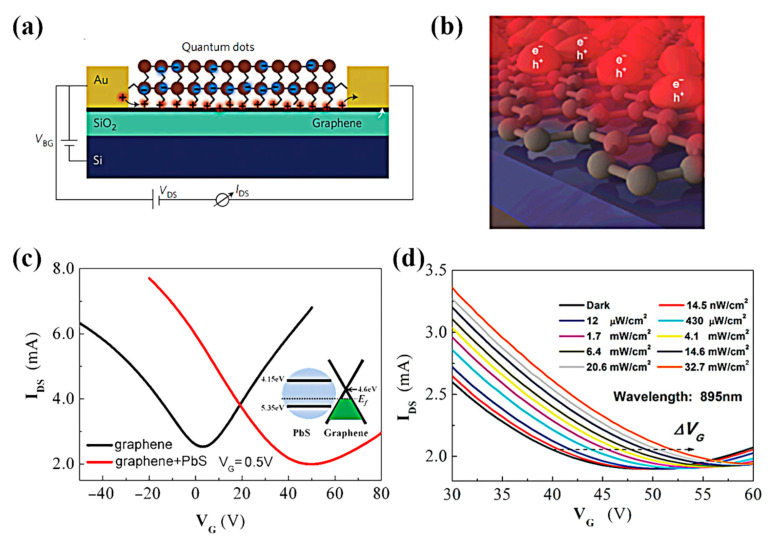
Photogating Effect. (**a**) Schematic of the graphene–quantum dot hybrid phototransistor. (**b**) Illustration of the interface charge distribution between nanoparticles and graphene. (**c**) Transfer characteristics of graphene transistors with or without the addition of PbS QDs on the graphene film. Inset: Energy diagram of the heterojunction of PbS QD and graphene. (**d**) Transfer characteristics of a PbS QDs/graphene transistor characterized under different light irradiation with the wavelengths of 895nm. (**a**) Reproduced with permission from [19]. Copyright Nature Publishing Group, 2012. (**b**) Reproduced with permission from [53]. Copyright Nature Publishing Group, 2017. (**c**,**d**) reproduced with permission from [52]. Copyright Wiley, 2012.

The PG effect (Figure 1b) is a particular case of the PC effect [49]. Thus, we usually think that the devices are photoconductive photodetectors or phototransistors [50]. The PG effect is thought to be a way of modulating conductivity by photo-induced gate voltages (V_GS_), rather than simply being attributed entirely to trap states [51,52]. The 2D material generates free electron–hole pairs in the presence of light (Figure 3a). If the electrons or holes are captured as trap states, the charged trap state can act as a local floating gate, strongly modulating the channel conductance. Consequently, the conductivity can be effectively modulated in this way. The positively charged hole trap state after photogenerated holes are trapped leads to electron doping. In this case, the transfer curve shifts in a negative direction (Figure 3c,d). The long lifetime of the photogenerated carriers leads to high gain due to the slow delocalization process. At the same time, the composite efficiency of the photogenerated carriers is greatly reduced, and the photocurrent of the photodetector does not quickly return to the dark current state even after the illumination stops, resulting in a deterioration of the high-frequency response performance of the detector. Moreover, traditional photoconductive-based detectors have a relatively large inherent bandgap of the sensitizing materials, so the working wavelength of this hybrid photodetector is limited to the visible and near-infrared. By coupling 2D material to the narrow bandgap semiconductor Ti_2_O_3_ (Figure 3b), photoexcited electrons are trapped in Ti_2_O_3_ when the incident light is absorbed by Ti_2_O_3_ nanoparticles, as demonstrated Yu et al. [53] The holes were transferred into the graphene channel and the charged Ti_2_O_3_ acted as a localized floating gate to modulate the channel conductance, enabling high responsivity of 300 A/W over a broadband wavelength range of up to 10 µm. The response time was estimated to be ~1.2 ms.

#### 2.1.3. Edge Effect

It is well known that in conventional 2D materials, electric fields are essential for separating photoexcited electrons and holes to generate photocurrents. However, in some 2D materials, the photothermoelectric effect also generates photocurrents after the temperature of the partial electrons is increased due to photoexcitation. The PV and PTE effects are usually present only in graphene with high doping levels [54,55]. In contrast, this edge effect dominates when graphene is completely undoped. This mechanism arises from the asymmetry of the electron–hole velocity in graphene [56]. For example, when electrons and holes are 0.1 eV away from the Dirac point, the difference in their intrinsic velocities is about 10^4^ m/s. This asymmetry means that when photons excite electrons and holes, they will diffuse, separate, and naturally generate local electric fields at different velocities. However, the diffusion is all around, and the local electric fields around the excitation point are circularly symmetric and cancel each other. As a result, there is no electric field in the circuit to drive the current. As shown in Figure 1c, when light hits the graphene edges, the diffusion is no longer symmetric circular, which creates a residual electric field that generates a photocurrent in the circuit [57]. Thus, graphene with well-designed edges can be used as a source of photocurrent. This mechanism is a vital feature because it dominates only undoped graphene, a phenomenon caused by a unique electron–electron scattering motion. This mechanism opens up possibilities for the design of graphene photodetectors, as it does not require any junction as a photocurrent source [58]. In the photoelectric and photothermoelectric effects, metal–graphene or graphene p–n junctions are crucial for electron–hole separation. With the edge mechanism, only the geometry of graphene is related to the photocurrent generation. In edge effect devices, there is no need to form a built-in electric field to separate the photogenerated carriers, which will significantly simplify the preparation process. The detector can totally turn off photocurrent generation by gate doping.

### 2.2. Thermal-Type Mechanism

#### 2.2.1. Photothermoelectric (PTE) Effect

**Figure 4:**
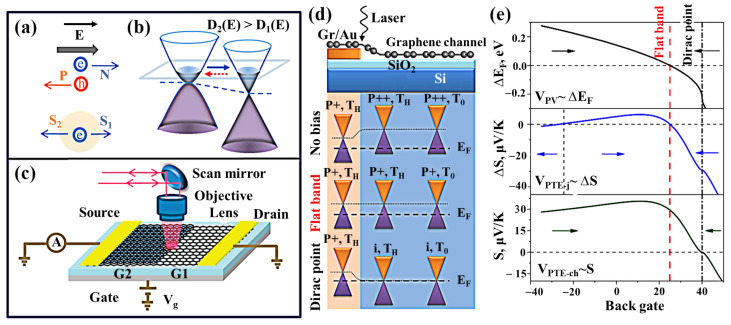
Photothermoelectric Effect. (**a**) In the top panel, the built-in electric field picture for photocurrent generation at a p–n junction. The direction of the field E is defined along the direction of electron movement. In the bottom panel, hot carrier diffusions at a material interface with different S1 and S2. (**b**) Aligned Fermi level of the bilayer (**left**) and single layer (**right**) graphene. D(E) is the density of states. The blue and red dashed arrows represent the electron flow direction induced by the built-in electric field and by the thermoelectric effect, respectively. (**c**) Schematics of the experimental setup and device geometry. (**d**) Schematic of a graphene/Au interface and associated band diagrams for various gating conditions. (**e**) Calculated gate voltage dependence of Fermi level difference (**top**) and Seebeck coefficient difference (**middle**) between the Gr/Au and Gr/SiO_2_ areas and gate voltage dependence of Seebeck coefficient for the graphene channel (**bottom**). (**a**–**c**) reproduced with permission from [48]. Copyright American Chemical Society, 2009. (**d**,**e**) reproduced with permission from [40]. Copyright Nature Publishing Group, 2018.

The PTE effect is caused by the light-induced temperature difference, which can cause thermal voltage and plays an essential role in the light response generation of many photosensitive 2D material devices (Figure 1d) [59,60,61,62]. Under illuminating conditions, hot carriers transfer energy slowly to the crystal lattice in the 2D material, forming a hot fermion distribution (Figure 4a–c). The PTE effect of photogenerated hot electrons can produce photovoltage (*V_PTE_*) as $V_{PTE}=\left(S_{1}-S_{2}\right)\Delta T$, where *S_1_* and *S_2_* are the Seebeck coefficients of 2D materials in two different doped regions, and ΔT is the electron temperature difference between the optical excitation area and the surroundings. According to the Mott formula, S is related to the conductivity of the material:

(1) $$S=-\frac{\pi^{2}k_{B}^{2}T_{e}}{3e}\frac{1}{\sigma }\frac{\partial \sigma }{\partial \varepsilon }$$

where $k_{B}$ is the Boltzmann constant, and ε is the energy that should be evaluated at the Fermi level, σ is the electrical conductivity. The PTE effect is understood to depend on the change in the Seebeck coefficient of the distribution of doping through 2D materials [63]. However, in the presence of an electronic temperature gradient, a second PTE effect may appear on a uniform graphene channel, that is, the establishment of a global electronic temperature difference through the device channel, which is called the PTE channel (PTE-ch) effect (Figure 4d) [40]. It can be seen in Figure 4e that V_PTE-ch_ should have a single sign change at the Dirac point of the graphene channel, which does not exist in PTE junction voltage (V_PTE-j_) or photovoltaic voltage (V_PV_). By using an asymmetric electrode contact device, one of the electrodes incorporates plasmonic nanostructures. Under the plasmonic excitation of metal nanostructures, the highly localized and enhanced electromagnetic fields around the nanostructures greatly improve the light absorption in nearby graphene, resulting in effective and localized carrier heating in graphene. The asymmetric plasma contact geometry generates a large electron temperature gradient across the entire graphene channel, thereby generating and observing a strong PTE-ch effect. The electronic response of PTE-based detectors is thermal electrons rather than lattice heating so that PTE-based detectors can achieve high bandwidth.

In photodetectors without bias voltage, the generation of photocurrent is related to PTE and PV effects. For a photodetector composed of a heterojunction, the direction of the photocurrent caused by the PTE and PV effects is the same, and the greatest photocurrent occurs when the light is spotted at the junction interface. Therefore, the two mechanisms are easily confused. To accurately identify the light response mechanism, we can distinguish the following principles. First, for PV detectors, the generation of a photocurrent requires that the incident photon energy be greater than the material bandgap. In contrast, for PTE detectors, which are based on thermal effects, the spectral response is not limited by the bandgap. Second, PV detectors rely on a built-in electric field and generate a nonlinear current-voltage (I–V) curve, while PTE detectors can operate on a linear current–voltage (I–V) curve. In PV photodetectors, the photoresponse is limited to near the junction interface. Whereas in PTE detectors, the spatial distribution of photocurrents can be extensive. Third, the distinction between the different mechanisms can also be based on whether the spectral response is wavelength dependent. When the absorbance of material is independent of wavelength, a PTE detector can be determined [64]. The disadvantage of PTE photodetectors compared to the PV photodetectors is the relatively long response time of phonon-dominated transport, typically in the order of milliseconds [65].

#### 2.2.2. Photobolometric (PB) Effect

**Figure 5:**
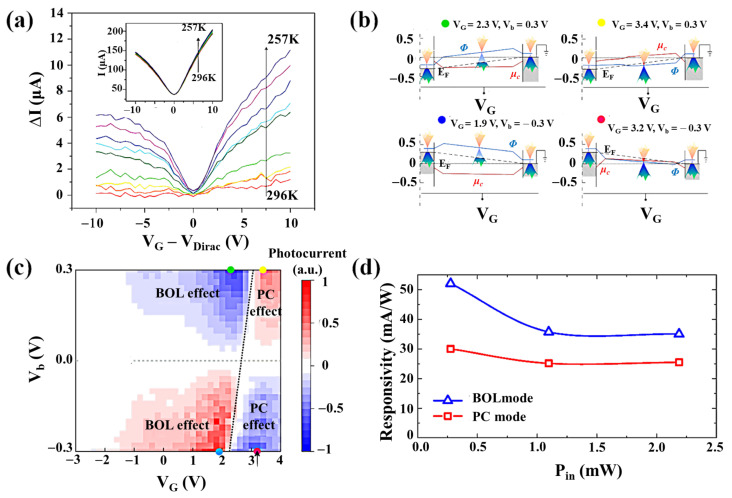
Photobolometric Effect. (**a**) Temperature-dependent change in current from its room temperature value as a function of gate voltage. Inset: gate voltage characteristic as a function of temperature when cooling down from room temperature. (**b**) Calculated energy band diagrams for the different cases. (**c**) Measured photocurrent map as the V_GS_ and the source–drain voltage (V_DS_) vary. (**d**) Measured responsivities with different input optical powers Pin. (**a**) Reproduced with permission from [69]. Copyright Nature Publishing Group, 2012. (**b**–**d**) reproduced with permission from [73]. Copyright Nature Publishing Group, 2019.

The bolometer detector is mainly made of semiconductor or superconductor absorbing material and is widely used in the submillimeter wave (THz) band [66,67,68]. It is one of the most sensitive detectors. The photobolometric effect (Figure 1e) is related to the direct heating of the 2D material by the incident photons, resulting in the change of the carrier mobility of the 2D material (Figure 5a) [69,70,71,72]. The sensitivity of the 2D material bolometer is determined by the thermal resistance R_h_ = dT/dP, where the bolometer causes a temperature increase dT by absorbing incident radiation (dP). The response speed of the detector is related to the specific heat capacity C_h_ of the 2D material. Since this photodetection mechanism is based on light-induced conductance changes rather than direct photocurrent generation, it requires an external bias. It can work on uniform 2D materials without the need of introducing the p–n junctions. An appropriate gate voltage can make the photobolometric effect the dominant mechanism of the phototransistor, and significantly improve the responsivity. In the silicon–graphene hybrid plasmon photodetector designed by Guo et al. [73], the PTE effect was the main mechanism of the photocurrent at zero bias voltage. Since the PTE photocurrent is generally insensitive to the V_DS_, when the V_DS_ was applied, the photocurrent greatly increased, which indicated that the PTE effect was no longer the main mechanism. The chemical potential of the graphene channel in the device is completely gate-controllable, and there is a transition region that gradually changed from the pinning region to the completely gate-controllable region. As shown in Figure 5b,c, when (V_GS_, V_DS_) = (2.3, 0.3) V and (1.9, −0.3) V, the graphene was highly doped. Therefore, the photobolometric coefficient became larger, and the photobolometric effect became the dominant mechanism (Figure 5d).

### 2.3. Topology (TP) Enhancement Mechanism

**Figure 6:**
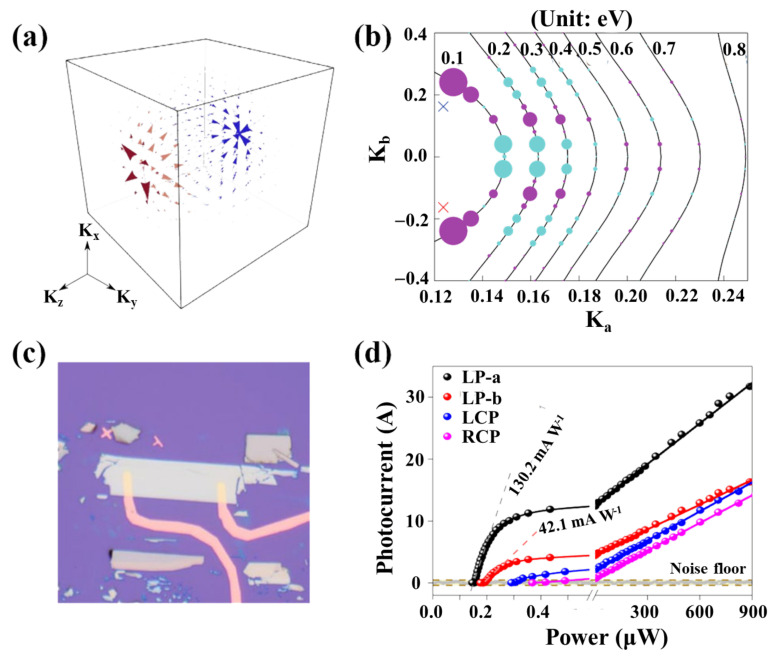
Topology enhancement effect. (**a**) Vector plot of the Berry curvature of Weyl semimetals in momentum space. (**b**) The integrand of the effective third-order optical conductivity tensor is shown along contours of fixed electron energy in momentum space (k_x_–k_y_) for TaIrTe_4_. (**c**) Optical microscopy image of the device. (**d**) Light-power dependence of the photocurrent at 4 μm incident wavelength in a FET based on TaIrTe4, a type-II Weyl semimetal. Reproduced with permission from [82,83].Copyright Nature Publishing Group, 2016, 2020.

Some topological properties of topological semimetals can greatly improve the light responsivity of the device [74,75,76,77,78]. In Weyl semimetal-based devices, these materials carry Weyl fermions that move parallel or anti-parallel to the spin moment, which defines the chirality of a specific Weyl cone (Figure 1f,g) [79,80]. The energy of Weyl fermions is proportional to their momentum, forming a cone structure in the energy–momentum space. The most important thing is that each chiral Weyl node can be regarded as the “monopole” of the Berry flux field (Figure 6a), the effective magnetic field in momentum space. These magnetic monopoles have a direct effect on the movement of electrons and cause various topological effects. One of the effects is on the offset current response, which is caused by the offset of the charge center of the non-centrosymmetric material excited by linearly polarized light during the inter-band optical excitation [81]. Compared with the semiconductor p–n junction, it constitutes a fundamentally different mechanism of generating the photocurrent. In the semiconductor p–n junction, the built-in electric field separates electrons and holes. The movement of the charge center can be expressed as the change in the Berry connection. The Berry connection is the vector potential that produces the Berry flux field and the velocity operator phase. Therefore, when the excitation occurs near the Weyl node where the Berry flux field diverges (Figure 6b), the corresponding conductivity tensor is expected to be significantly enhanced. This topology enhancement effect has recently been experimentally verified.

Ma et al. used the inherent topological properties of Weyl’s semimetallic TaIrTe_4_ to greatly improve the responsivity of the detector in the mid-infrared band (Figure 6c), breaking through the long-term technical bottleneck of the responsivity of the semimetallic detector [83]. Ma et al.’s work mainly used the divergent Berry curvature of the Weyl semimetal near the Weyl point, so that the displacement current response related to the Berry field was significantly enhanced near the Weyl point. The transition caused by the lower energy photon will be closer to the Weyl point. However, the lack of external voltage bias and the influence of topology effects require special considerations in device design. No external bias means that the turn-on threshold power of the field-effect transistor photodetector must be minimized to increase its sensitivity (Figure 6d), which requires the use of conventional methods to reduce the semimetal contact barrier, such as semimetal doping or selecting a suitable work function metal contact.

## 3. Performance Parameters

To better compare the performance of photodetectors of different sizes and under different operating conditions, we summarize a list of key metrics commonly used to describe the performance of photodetectors, including responsivity, quantum efficiency, signal-to-noise ratio, bandwidth, and detection capability.

### 3.1. Responsivity (R)

The ratio of the photocurrent (*I_p_*) magnitude to the incident photo power (*P*) is defined as the spectral responsivity and expressed as:

(2) $$R=\frac{I_{p}}{P}=\frac{I_{light}-I_{dark}}{P}$$

where *I_light_* and *I_dark_* are the currents measured in the illuminated and dark environments, respectively. In general, the responsivity varies with the incident optical power, wavelength, and the applied electric field. Commercially available silicon-based photodiodes can reach 500 mA/W at the sensing wavelength of 405–1100 nm [25], whereas the graphene responsivity is about 10 mA/W [5]. Combined with the quantum dots such as Cu_2_O, the graphene photodetectors can reach a responsivity of 10^10^ A/W [39]. 

### 3.2. External Quantum Efficiency (EQE)

When the phototransistor operates based on the PV effect, not all the incident photons can be absorbed to generate electron–hole pairs. Even the electron–hole pairs are generated, some of them cannot contribute to the photocurrent due to recombination or capture processes. Therefore, EQE is defined as:

(3) EQE=IpePhv=Rhceλ

where *e* is the electron charge, *h* is the Planck constant, *c* is the speed of light, *λ* is the wavelength of light, and v is the frequency of light. Due to the low light absorption efficiency of the monolayer 2D material, a higher *EQE* can be achieved by increasing the thickness of the material to improve the light absorption efficiency and reduce the recombination of photogenerated carriers.

### 3.3. Internal Quantum Efficiency (IQE)

In a comparison with *EQE*, *IQE* is the ratio of the number of electron–hole pairs produced to the number of photons absorbed, which is rewritten as:

(4) $$IQE=\frac{EQE}{total\;photon\;absorption}$$

In general, *IQE* is always greater than *EQE* because refraction and transmission cannot be eliminated. In addition, if the material is extremely thin, the interference effect of light needs to be considered.

### 3.4. Response Time (τ) and Bandwidth (B)

Response time of the photodetector reflects the ability to detect a rapidly modulated light signal. It includes the rise time $\tau_{r}$ and the fall time $\tau_{f}$ which are the time required for the peak current of the device to flow from 10 to 90% and the time required for the peak current of the device to flow from 90 to 10%, respectively. In most cases, there cannot have a balance between excellent response rate and response time. Commercially available silicon photodiodes typically have a rise time of 50 ns [25], while graphene photodetectors can have a response time of several hundred picoseconds [40]. Conductivity and photoconductivity usually change the response time of the photodetector. Due to the trap states, the attenuation of the photocurrent is also strongly dependent on the intensity of the light incident on the device. The longer response time may be attributed to the low conductivity and traps in the 2D material film. Since the trap density, trap energy distribution, and carrier capture probability may be different, more complex mechanisms are at play and deserve further investigation.

For most optoelectronic devices, the optical responsivity depends on the optical modulation frequency (*f*) and is expressed as:

(5) $$R\left(f\right)=\frac{R_{0}}{\sqrt{1+{\left(2\pi f\tau \right)}^{2}}}$$

where *R_0_* is photoresponsivity measured under static illumination. As f increases, *R* decreases. The modulation frequency at which the optical responsivity decreases to −3 dB is called the bandwidth, also known as the cutoff frequency. The broadband photodetectors are needed for high-speed information transmission, and the graphene photodetectors can have a bandwidth of about tens of GHz [40].

### 3.5. Signal-to-Noise Ratio (SNR)

Since noise produces random fluctuations in the output of the detector signal, the presence of noise in the detection process can have an impact on the detection of the signal. *SNR* is given as:

(6) $$SNR=\frac{Signal\;power}{Noise\;power}$$

The signal power can be detected only when it is higher than the noise power, i.e., *SNR* > 1.

### 3.6. Noise Equivalent Power (NEP)

*NEP* is the minimum optical signal power that a photodetector can detect or distinguish from the total noise. It is defined as the optical input power required to achieve an *SNR* of 1 at a bandwidth of 1 Hz and can be expressed as:

(7) $$NEP=\frac{P}{\sqrt{B}}$$

where *P* is the incident power that results in *SNR* = 1. *NEP* in commercially available silicon photodiodes can reach 10^−14^ W/Hz^−1/2^ [18], whereas in graphene photodetectors it is about 10^−12^ W/Hz^−1/2^, mainly because of the high dark current of graphene [84].

### 3.7. Detectivity ($D^{*}$)

To better compare the performance between different detectors, the effects of bandwidth, geometry, and device area should be considered. The detectivity reflects the sensitivity of the photodetector, and it takes into account the *NEP*, area, and bandwidth as:

(8) $$\;D^{*}=\frac{{\left(AB\right)}^{\frac{1}{2}}}{NEP}$$

where *A* is the photosensitive area. If the dark current of the device is much larger than the noise, the detectivity can be further expressed as:

(9) $$D^{*}=\frac{RA^{\frac{1}{2}}}{{\left(2eI_{dark}\right)}^{\frac{1}{2}}}$$

A higher detectivity indicates better detection performance of the photodetector, which can be improved by increasing the response rate, increasing the detection area, and reducing the dark current of the device. The detectivity of silicon photodiodes is about 10^10^ Jones in the visible range [25], while 2D materials under 405 nm illumination can reach 10^16^ Jones by combining with quantum dots to form a hybrid structure [42]. The detectivity of differently structured phototransistors is summarized in Table 1.

## 4. Photocurrent-Enhanced Structure of 2D Materials

Some 2D materials (such as graphene, BP, and InSe [18]) have the characteristics of high carrier mobility and high Fermi velocity that can be fully utilized, and thus have excellent high-frequency response characteristics, which are mainly used for ultra-fast optical detection applications such as optical communications and optical modulators. Although it is possible to adopt a multilayer structure or adjust the bandgap through the V_GS_ to improve the light absorption of the 2D material, it is still difficult to fundamentally solve the problem of low photoelectric response caused by weak light absorption. Therefore, to enhance the light response of the photodetector, multiple types of photodetector structures have been developed.

**Figure 7:**
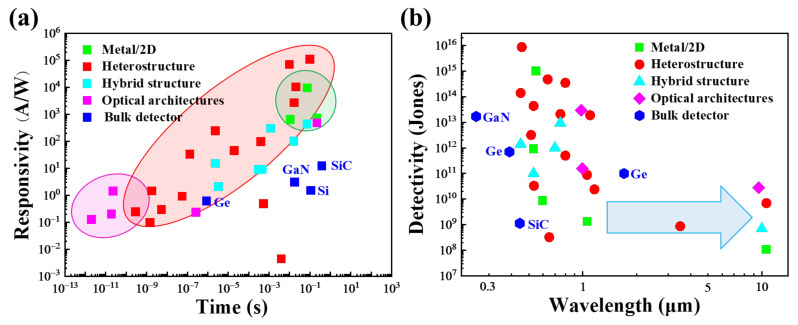
(**a**) Comparison of the responsivity and response time of 2D material detectors. (**b**) Specific detectivity versus response wavelength in different structures photodetectors.

Many approaches, such as the construction of metal/2D structures, vdWs heterostructures, hybrid structures, and optical architectures, are useful for improving device performance (Figure 7a) [119,120]. In terms of optical architecture (cavities, waveguides, and plasmonics) integration, the plasmon structure enhances light absorption in 2D materials by enhancing local electromagnetic fields and subwavelength scattering, thereby enhancing the coupling of 2D materials with light. However, this architecture suffers from plasmonic losses due to energy dissipation. Moreover, the plasmon structure is bonded to the surface of the 2D material, and the local surface plasmon resonance (LSPR) frequency is fixed, resulting in narrow band enhancement. The optical resonant cavity structure can form a standing wave cavity resonator for light waves. It can achieve effective light confinement with a high-quality factor in space, resulting in a strong interaction between light and matter, but it is not suitable for broadband applications. The structure of 2D material and waveguide integration is particularly suitable for high-speed applications at the telecom wavelength, but the on/off ratio of the photodetector is not sufficiently high. To obtain high responsivity, high-mobility 2D materials can be combined with 2D materials with different band gaps or quantum dots to form heterostructures. Heterojunction photodetectors have distinct photovoltaic properties, which give them excellent photoresponse performance and self-powered photodetection characteristics. In addition, their special type of heterostructure can facilitate efficient separation and transfer of photogenerated electron–hole pairs. However, the construction of heterojunction photodetectors is limited by the type of material and requires the search for semiconductors with matching band structures.

The photodetector can be further optimized by employing a rational design and selecting the right materials, including high-quality 2D materials, metals for contacts, and semiconductor materials modified on the channel. A long carrier lifetime is required to obtain maximum responsivity. By causing defects in 2D materials, the carrier lifetime can be effectively extended. However, this approach comes at the cost of response time. For the sensitizer, a high absorption coefficient, large spectral coverage, and desirable tunable spectral coverage are prerequisites. In addition, to facilitate efficient charge transfer to the transistor channel and thus obtain high quantum efficiency, the sensitizer layer should also have good electronic properties in terms of carrier diffusion length, carrier mobility, doping, and minority carrier lifetime. For future practical applications, 2D materials should have a large area, high quality, and uniform growth. For photodetectors, further improvements are needed to improve material stability and material light absorption and to reduce dark currents and lower response times. The compatibility of 2D materials with existing manufacturing processes is also an issue. It is necessary to take full advantage of their thinness and their physical nature of interaction with light and to propose suitable photodetection mechanisms to achieve an excellent performance of 2D material photodetectors.

From Figure 7b we can clearly see that the detectivity of 2D photodetectors in the visible range is comparable to or better than that of bulk photodetectors. That mainly because metal/2D structures, heterostructures, hybrid structures and optical architectures can be easily form in 2D photodetectors but not in bulk photodetectors. Dark currents are typically suppressed better in metal/2D structures and heterostructures. Hybrid structures achieve significant gains benefiting charge trapping. High light absorption can be obtained from the designed Optical architectures. Due to the limitation of the band gap of the material, it is difficult for the photodetector to cover the broad-spectrum detection. Photodetectors made of 2D materials and other materials can combine the advantages of both materials to achieve a broadband response with a high absorption coefficient, large spectral coverage, and ideal tunable spectra, and the recent emergence of gapless semimetals materials can extend the detection range to Long-Wave Infrared. By taking advantage of the shifted current response of the semimetal, the responsivity will be improved by at least two orders of magnitude. Although micro-photonic devices based on 2D materials still have limitations, they have great potential in many applications such as images and photon energy conversation using heterostructures.

### 4.1. Metal/2D Material Structures

**Figure 8:**
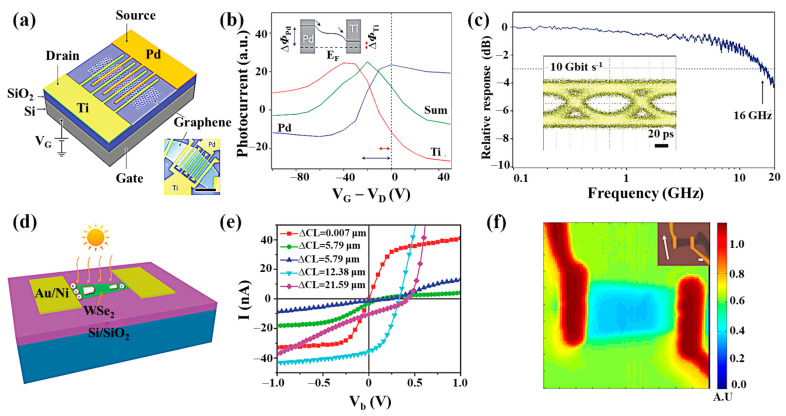
Metal/2D material structures. (**a**) Metal–graphene–metal (MGM) photodetectors with asymmetric metal contacts. (**b**) The dotted line denotes the Fermi level. $\Delta \phi_{Pd}$ and $\Delta \phi_{Ti}$ represent the difference between the Dirac point energy and the Fermi level in palladium- and titanium-doped graphenes, respectively. (**c**) Relative photoresponse versus light intensity modulation frequency. The -3dB bandwidth of this MGM photodetector is 16 GHz. Inset: receiver eye-diagram obtained using this MGM photodetector, showing a completely open eye. (**d**) Photodetectors with asymmetric contact geometries. (**e**) Photodetectors with different degrees of asymmetries. (**f**) Weyl semimetal scanning photocurrent response. (**a**–**c**) reproduced with permission from [5]. Copyright Nature Publishing Group, 2010. (**d**,**e**) reproduced with permission from [90]. Copyright Wiley 2019. (**f**) Reproduced with permission from [91]. Copyright Nature Publishing Group, 2019.

Photodetectors based on metal/2D material contact were the first to be studied [44]. The increased optical responsivity of photodetectors with metal/2D structures can be achieved due to the Schottky barrier in the contact area [87,88,89], [121,122,123]. The Schottky barrier limits the transmission of dark currents [124,125]. At the same time, the Schottky built-in electric field allows for the more efficient separation of photogenerated carriers [126,127]. Consequently, enhancing the built-in electric field is an effective way to improve the detectivity and separation efficiency of photogenerated carriers and promote the enhancement of photocurrents [128,129]. In earlier reports, the photocurrent was generated by the local illumination of the metal/graphene interface of the back-gate graphene field-effect transistor [54,130]. The current generated is due to the PV effect. In 2009, Xia et al. fabricated the first graphene photodetector using mechanically exfoliated graphene and investigated the photocurrent generation mechanism of metal/graphene contact using high-resolution photocurrent imaging by near-field scanning optical microscopy with an FET-structured device [45]. The conclusion was that the photocurrent generation is related to the energy band bending at the metal/graphene contact interface due to different work functions and that the magnitude and direction of the photocurrent can be controlled by changing the position of the Fermi energy level through an applied gate voltage. Most of the electron–hole pairs are generated in graphene when both electrodes are illuminated in the vicinity of the light due to the same metal contact generating an internal electric field of equal size and opposite direction, leading to carrier recombination without any contribution to the external photocurrent. Photocurrents only occur in the sub-micron width region of the metal/graphene interface. It is impractical to disrupt the mirror symmetry of the device with bias because the semimetallic nature of graphene generates large dark currents. Therefore, two approaches have been proposed to break the symmetry of the photodetector [131]. The first method is to select contact electrodes with different work functions, which leads to a difference in Schottky barrier height and a different depletion layer width between the two contact junctions. By using graphene as a channel material, T. Mueller et al. proposed an improved graphene photodetector with an asymmetric metallization structure to increase the effective light detection area (Figure 8a) [5]. They demonstrated that an asymmetric metallization scheme can break the mirror symmetry of the potential profile built into the channel, with two electrodes made of palladium and titanium, respectively. Doping under these two electrodes is different, and the polarities of these two electrodes are opposite at different V_GS_. Therefore, when a suitable V_GS_ is selected, the photocurrent near the two electrodes can flow in the same direction, leading to an overall photocurrent enhancement (Figure 8b). Error-free detection is achieved in a 10 Gbit/s optical data link with a wavelength of 1.55 mm (Figure 8c). However, due to the semimetallic nature of graphene, the response is fast but the dark current is large. The second approach is to exploit the geometric asymmetric contact effect, which means that the difference in contact area or contact length between two junctions causes a non-zero photocurrent. Zhou et al. demonstrated a self-driven photodetector based on a multilayer WSe_2_ sheet (Figure 8d) [90]. Due to the Fermi energy level of WSe_2_, which lies midway between the conduction band minimum (CBM) and valence band maximum (VBM), Ni was chosen as the contact electrode to obtain a high Schottky barrier for low dark currents. The metal/2D photodetector with asymmetric geometrical contacts shows an apparent PV effect. The high responsivity of 2.41 A/W can be obtained at zero external bias. In combination with the ultra-low dark current, a high detectivity of 9.16 × 10^11^ Jones is achieved (Figure 8e). Metal electrodes with high work function have been fabricated to provide a larger built-in electric field and improve the responsivity of the device. However, the improvement of the built-in electric field is limited by the small tuning range of the metal work function [85,86]. Moreover, variations in the electrode material increase the risk of instability and complexity in the fabrication process. Therefore, a method to improve the strength of the built-in electric field in a stable electrode material should be implemented. Furthermore, most of the metal/2D structured devices are fabricated based on graphene. They have relatively high dark currents but show feasibility for high-speed light detection applications over a wide wavelength range. Recently, topological semimetals Cd_3_As_2_, Td-MoTe_2_, and Td-WTe_2_ have shown great advantages in the wide wavelength range (Figure 8f) [91,132,133]. Although the responsivity of semimetallic-based detectors is generally not optimal, their bandgap-free electronic structure gives them an unprecedented broadband optical response. There is no energy gap in the semimetals to limit the energy of detectable photons, so the range of detectable photon energies can be extended to the low energy end. In the absence of a bandgap, however, the brief lifetime of the photoexcited carriers is greatly reduced by fast electron–electron scattering, thereby increasing the speed of semiconductor operation.

In comparison, the responsivity of the metal/2D material structure is still low. In addition to the symmetrical electrodes causing the photocurrent to be zero at V_DS_ = 0 V, the low responsivity is also due to the low absorbance of the semiconductor, the short existence time of photogenerated carriers, and the small effective detection area. Therefore, we can increase the photocurrent by selecting the appropriate material thickness to achieve high light absorption efficiency. In addition, the carrier lifetime can be effectively extended by causing defects in the 2D material. However, it will affect the device response speed.

### 4.2. 2D Material Heterostructure

**Figure 9:**
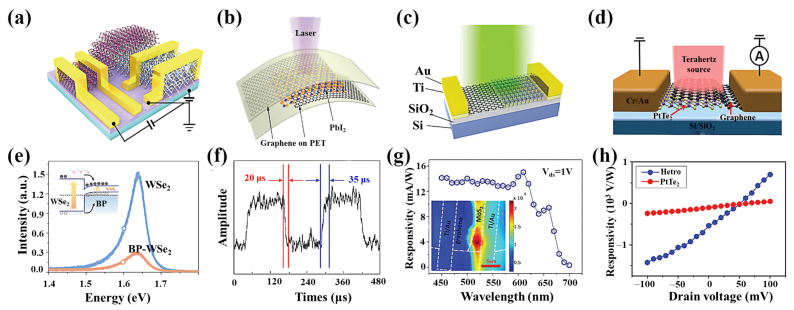
Heterostructure photodetector. (**a**) BP–WSe_2_ heterostructures schematic. Schematic band diagram of BP–WSe_2_ heterostructures. (**b**) schematic of photoelectric measurement of the flexible device with near-ultraviolet laser source. (**c**) Schematic of lateral graphene–MoS_2_ heterostructure Schottky photodetector under the illumination. (**d**) Schematic representation of the electrical configuration for the bow-tie-type PtTe_2_-based THz detector. (**e**) PL spectra of monolayer WSe_2_ and the BP–WSe_2_ heterostructure under an incident laser. Inset: Schematic band diagram of BP–WSe_2_ heterostructures. The dashed line denotes the Fermi energy of BP and WSe_2_. (**f**) Fast device photoresponse, giving the rise and decay time, respectively. (**g**) Spectral responsivity for the detector. The absorption for the light of the device is mainly limited to the bandwidth of MoS_2_, corresponding to the test in the inset. Inset: scanning photocurrent mapping of the detector. (**h**) The voltage responsivity of the two devices under different biases at 0.12 THz. (**a**,**e**) reproduced with permission from [10]. Copyright Nature Publishing Group, 2019. (**b**–**d**,**f**–**h**) reproduced with permission from [94,98,102]. Copyright Wiley, 2018,2019.

Planar connections between different 2D materials and composite semiconductors can be used as Schottky photodiodes to improve light detection [96,97,103]. Zong et al. proposed the BP-WSe_2_ heterostructure (Figure 9a), and by characterizing its photoluminescence (PL), it was shown that the heterostructure forms a type-I energy band alignment and achieves an efficient energy transfer from WSe_2_ to thin-film BP (Figure 9e) [10]. The enhancement of the PL signal is attributed to the light absorption by the monolayer WSe_2_, with higher absorption resulting in more electron–hole pairs, which leads to a larger enhancement factor. These results suggest that the efficient electron transfer in the heterostructure can be used to a greater advantage in the photodetector. Photodetector configurations combining TMDs as light-interacting materials and graphene as electrodes can effectively improve the photodetection performance [134]. Moreover, recent studies have shown that vertical heterogeneous structures based on graphene/TMDs can further improve the performance of photoelectric detection, yielding high optical responses, larger quantum efficiencies, and shorter response times. The electrical characteristics of these devices exhibit rectification, and their barrier energy depends on the semiconductor material. For low dark currents, the semiconductor–graphene photodetectors operate with reverse bias. Light absorption occurs in semiconductors and graphene acts as a charge-carrier transport channel. Under illumination, the photogenerated carriers in the heterojunction are spatially separated under the junction’s internal built-in field, and electrons or holes are transferred to the channel region. Due to diffusion in their channel, the presence of these carriers effectively modulates the conductivity of the graphene. By applying V_DS_, the carriers drift and recirculate in the graphene channel. However, they also have low responsivity due to the lack of an optical gain mechanism or due to the persistent photoconductivity, which is not suitable for the photodetectors. Besides the typical bilayer vdW heterostructures, photodetectors based on 2D material sandwich structures have been extensively studied [101]. Usually, highly absorbent 2D materials are used as absorbents. The detectors are based on vertically stacked PbI_2_/graphene heterostructures, using graphene as the transparent electrode material and PbI_2_ as the light-absorbing substance (Figure 9b) [98]. The sandwich structure of the device exhibits high sensitivity, a short response time (Figure 9f), and high-resolution imaging capability. Furthermore, photodetectors based on 2D material heterojunctions with 3D (bulk) semiconductors have been extensively investigated in recent years. These include graphene/Si photodetectors and deep-UV photodetectors based on MoS_2_/p-GaN heterojunctions [135,136]. A built-in electric field will appear near the 2D/3D structural interface, limiting the recombination of photogenerated carriers. This provides a new path for designing fast response devices.

Traditional semiconductor Schottky photodetectors are currently widely used in commercial photodetectors. The current key issue is constructing a 2D material heterostructure photodetector with high detectability and large-scale manufacturing capabilities [137]. Deng et al. fabricated transverse graphene/MoS_2_ Schottky junctions (Figure 9c), which benefited from strong absorption of light, efficient separation of photoexcited carriers, and fast charge transport in Schottky junction devices with responsivity up to 105 A/W (Figure 9g) [94]. The heterostructure photodetector array has also been proven, showing large-scale manufacturing capabilities. These results demonstrate the potential of two-dimensional material heterojunction devices in optoelectronic devices. With the rapid development of the modern semiconductor industry, unpowered or self-powered devices have become an indispensable part of electronic components and optoelectronic products [131,138]. Epitaxially connected TMDs transverse heterojunctions with maximum built-in potential at the heterojunction interface can rapidly separate light-generated electron–hole pairs without external bias. Wu et al. reported a PV photodetector based on a MoS_2_/WS_2_ flat heterostructure [95]. This detector exhibits a responsivity of 4.36 mA/W and a detectivity of 4.36 × 10^13^ Jones.

While conventional heterojunction photodetectors show great advantages, they have difficulty covering a broad spectrum in terms of spectral response due to bandgap limitations [139,140,141,142]. Huang et al. developed a PtTe_2_/graphene heterostructured photodetector (Figure 9d) [102]. PtTe_2_ is a type II Dirac semimetal with a bulk cone and a topologically protected type II Dirac surface state. Theoretically, the symmetric inverse monopole of the Dirac cone produces precisely the opposite photocurrent, so that the photocurrent should be zero for all Dirac cones. However, the PtTe_2_-based device shows excellent photo responsivity at zero bias (Figure 9h), the mechanism of which needs to be further investigated. When bonded to graphene through weak vdW interactions, the excess flow of non-equilibrium carriers can move more directionally, exhibiting a responsivity of more than 1.4 kV/W in the THz band due to the built-in field between graphene and PtTe_2_. Therefore, topological semimetals may be ideal materials for implementation in high-performance photodetection systems in the THz band.

### 4.3. 2D Material Hybrid Structure

**Figure 10:**
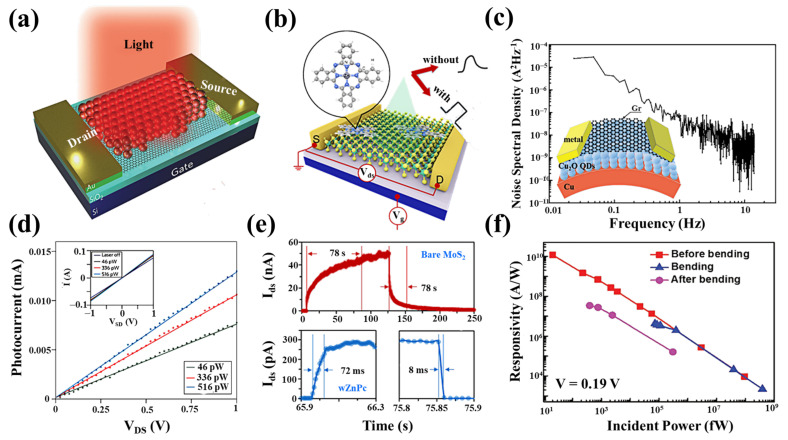
Hybrid structure. (**a**)Schematic of the graphene–quantum dot hybrid phototransistor, in which a graphene flake is deposited onto a Si/SiO_2_ structure and coated with PbS quantum dots. (**b**) Schematic illustration of the field-effect transistor based on ZnPc-treated MoS_2_. (**c**) Noise spectral density of the photodetector. Inset: Illustration of the bent photodetector. (**d**) Photocurrent of the graphene–quantum dot transistor for different optical powers as a function of VDS showing a linear dependence on the bias. Inset: total current. (**e**) The time-dependent photoresponse dynamics for a MoS_2_ device after varied ZnPc treatments are plotted on a linear scale. (**f**) Responsivity versus incident power for the photodetector before bending, bending with a curvature of 40 mm and after bending. (**a**,**d**) Reproduced with permission from [19]. Copyright Nature Publishing Group, 2012. (**b**,**e**) Reproduced with permission from [104]. Copyright American Chemical Society, 2018. (**c**,**f**) Reproduced with permission from [39]. Copyright Wiley, 2018.

A common obstacle for 2D materials is the limited absorption due to their atomically thin profiles and limited spectral selectivity, which is determined by the bandgap of the material [93,99,143]. Hybrid graphene/quantum-dot (QD) phototransistors are reported to exhibit high gain and optical sensitivity determined by the size of the QDs [52]. Integrating QDs with 2D materials has several advantages. First, 2D materials are less light-absorbing, whereas thicker QDs can use light efficiently [144,145]. Second, the 2D material as a channel can avoid the problem of low mobility. Third, the broadband absorption of the QDs can compensate for the limited response band of some 2D materials. G. Konstantatos et al. [19] demonstrated a notable device structure based on the PbS QDs/graphene heterojunction (Figure 10a). The optical responsivity of the device can be as high as 10^7^ A/W by taking advantage of the superior light absorption properties of PbS and the formation of an optical gain mechanism (Figure 10d). The sensing mechanism of PbS QDs/graphene phototransistors can be attributed to the charge transfer between PbS QDs and graphene. Electron–hole pairs are generated under light irradiation in PbS QDs, and the generated electrons and holes will be transferred to a place with a lower energy level. Since electrons and holes are transferred at different rates, the number of electrons and holes in the QD will also be different, which will result in a net negative or positive charge in the QD. The transfer curve shifts horizontally to a positive voltage under illumination, indicating that the QD has a net negative charge, attracting more holes in the graphene film. In other words, due to the net negative charge in the QD, a higher V_GS_ is required to obtain a charge-neutral point (Dirac point) in the graphene transistor.

Flexible, high-sensitivity photodetectors are ideal for the future of wearable optoelectronic devices. Graphene is promising due to its excellent electrical, optical, and mechanical properties, which make it extremely attractive for flexible optoelectronics and light detection applications. In 2019, Liu et al. proposed an internal current gain mechanism based on the 2D–0D light detection system (Figure 10c) [39]. They developed a flexible non-transferred hybrid graphene/Cu_2_O QDs photodetector, which can reach a detectivity of 10^10^ A/W. After bending, the responsivity of the photodetector still reaches 10^6^ A/W (Figure 10f), which paves the way for graphene to be applied to high-sensitivity flexible optoelectronic devices. The 1/f noise dominates at low frequencies in graphene/Cu_2_O QDs photodetectors and is significantly higher than both the shot noise and the thermal noise (Figure 10c). The carrier trapping and detrapping processes caused by the graphene/Cu_2_O QDs interface states cause the 1/f noise at low frequencies, and defects or disorders exist in the graphene sheet. In 2D material devices, flicker (1/f) noise is a major contributor to the low-frequency noise, and it increases as the reciprocal of the device area. Because of their high surface-to-volume ratio, 2D materials are extremely sensitive to their surroundings. This enables the detection of environmental chemicals such as methanol and ammonia using 1/f noise measurements in 2D FET. When the vapors of these chemicals are exposed to the 2D FET, both the channel resistance and the noise amplitude increase. However, the change in resistance is negligible, whereas the change in noise amplitude is 1500%. Noise-based sensors have a much shorter reset time than resistance-based sensors, which gives them a significant advantage in gas sensing applications [146,147]. However, reported 2D–0D hybrid devices use toxic nanomaterials as sensitization layers, which may limit their practical applications. Kwak et al. first fabricated 2D–0D hybrid photodetectors using non-toxic InP QDs as the light-absorbing layer and BP as the transport layer [42]. Furthermore, to remove the long surface ligands of the colloidal InP QDs, the prepared hybrid devices were chemically treated with 1,2-ethanedithiol (EDT) for layer-by-layer deposition. A high optical response of up to 10^9^ A/W and a high detectivity of 4.5 × 10^16^ Jones were produced with a 405 nm laser.

However, devices with integrated QDs are susceptible to surface trap states and stability problems. The limitation of carriers in QDs hinders their application as photodetectors. Huang et al. achieved ultrafast photoresponse kinetics by the surface assembly of the organic molecule ZnPc in a monolayer MoS_2_ detector (Figure 10b) [104]. They found that the assembly of ZnPc molecules tends to compensate for the intrinsic electron doping in MoS_2_ by withdrawing electrons from the MoS_2_. This charge transfer causes spontaneous reverse separation of electron–hole pairs under illumination, thereby driving the holes onto the ZnPc molecules, which nicely suppresses the rather slow capture of a few carriers into the intrinsic trap state in MoS_2_ and the substrate, providing a faster response compared to pure MoS_2_ (Figure 10e). Zhang et al. propose a hybrid TMD–QD combination that combines the strong light gathering of QDs with the high carrier mobility of TMDs, paving the way for the next generation of photodetectors [105].

### 4.4. Optical Architectures (Cavities, Waveguides, and Plasmonics) Photodetectors

Single-layer graphene (SLG), a typical representative of 2D materials, absorbs 2.3% of the incident light, which is high for atomically thin materials. For flexible and transparent optoelectronic devices, this is an attractive property. However, there is an urgent need to enhance the light absorption of 2D materials for some applications.

**Figure 11:**
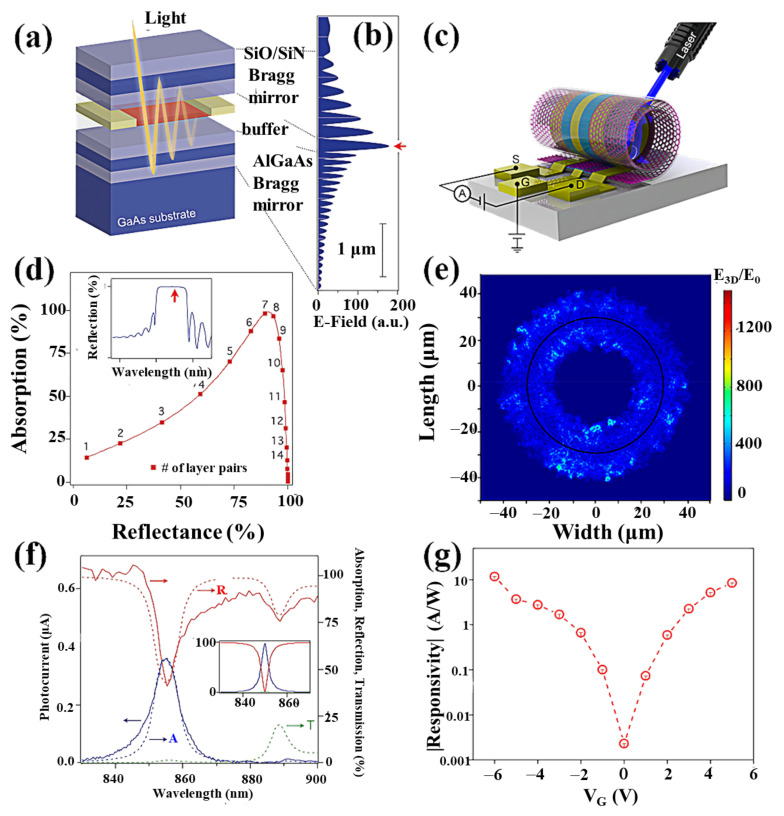
Optical microcavities. (**a**) Schematic drawing of a graphene microcavity photodetector. Distributed Bragg mirrors form a high-finesse optical cavity. The incident light is trapped in the cavity and passes multiple times through the graphene. (**b**) Electric field amplitude inside the cavity. (**c**) Schematic diagrams of the 3D GFETs after the roll-up. (**d**) Calculated dependence of optical absorption in a single layer graphene sheet on the reflectivity of the top mirror. (**e**) The simulated distribution of the electric field magnitude near a 3D GFET with one rolled-up winding. (**f**) Spectral response of the single-layer graphene device. The dashed lines show calculation results: reflection R (**red**), transmission T (**green**), and absorption A (**blue**). The solid lines are measurement results: reflection (**red**), photocurrent (**blue**). Inset: Theoretical result for normal incidence light. (**g**) The gate-voltage-dependent responsivity of the 3D GFETs under V_DS_ = 0 V. Reprinted with permission from [107,148]. Copyright American Chemical Society, 2012,2018.

One method to enhance the absorption is based on the integration into optical microcavities or planar photonic crystal cavities first proposed by M. Furchi et al. in 2012, which integrates planar optical microcavities into graphene transistors (Figure 11a,b) [148]. The top and bottom mirrors are made of two metal materials, Ag and Au, with graphene placed in the middle of the cavity. This structure can increase the photocurrent by about 20 times (Figure 11d,f). The reflector absorbs the incident light, which increases additional loss. M. Engel et al. reported a resonant cavity with a distributed Bragg reflector consisting of alternating layers of material one-quarter wavelength thick, with Si_3_N_4_ as the buffer layer in the cavity and graphene located at the point of maximum optical field intensity in the cavity, where incident light is captured in the planar cavity [149]. They passed through the SLG several times, thus enhancing the absorption capacity to 60% light absorption and 21 mA/W responsivity. The main disadvantage of this planar microcavity integrated graphene photodetector is that the optical responsivity can only be increased at the designed resonant frequency. In order to solve this problem, a self-rolling technique is used to fabricate a three-dimensional tubular optical microcavity. Deng et al. fabricated self-rolled-up 3D graphene field-effect transistor (GFET) photodetectors (Figure 11c), which have shown for the first time a balance among the optical responsivity, spectral range, and bandwidth [107]. The self-rolled-up 3D GFETs provide a naturally resonant microcavity that enhances the optical field and increases the area of the light–graphene interaction, thus significantly enhancing the light absorption (Figure 11e) [108]. The optical responsivity of the 3D GFETs is significantly improved. At the same time, the inherent ultra-fast and ultra-broadband optoelectronic properties of graphene are maintained (Figure 11g).

**Figure 12:**
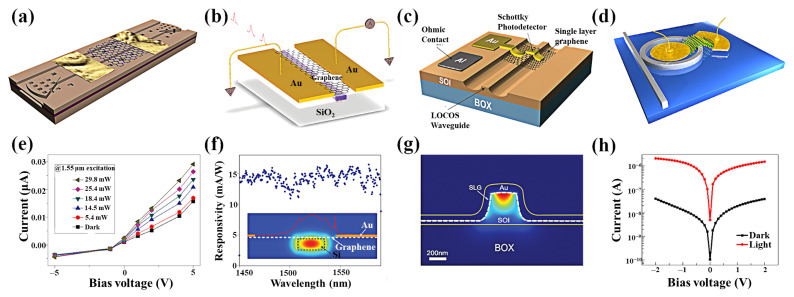
Optical waveguides. (**a**) Schematic of the graphene/silicon-heterostructure waveguide photodetectors. (**b**) Schematic of the device. The silicon bus waveguide fabricated on a silicon-on-insulator wafer is planarized using SiO2. (**c**) Schematic of Si-SLG Schottky photodetectors integrated with photonic waveguides. (**d**) A schematic of an MRR-integrated MoTe_2_ photodetector. (**e**) Dark and illuminated current versus bias voltage of the same device as under different incident light powers. (**f**) Broadband uniform responsivity over a wavelength range from 1450 nm to 1590 nm at zero bias. Inset: simulated electric field of the TE waveguide mode. (**g**) Finite element simulated optical intensity profile of an SPP waveguide mode supported by an M-SLG-Si structure. (**h**) Typical I–V characteristics (semi-logarithmic plot) of the Au/MoTe_2_/Au diode showing around two orders of magnitude enhancement for light (red) over dark (black) conditions. (**a**,**b**,**d**–**f**,**h**) Reproduced with permission from [111,152,153]. Copyright Nature Publishing Group, 2013, 2019. (**c**,**g**) Reproduced with permission from [154]. Copyright American Chemical Society, 2016.

Another approach involves the coplanar integration of 2D materials with optical waveguides [150]. The use of in-plane evanescent waves is considered an effective technique for enhancing the interaction of light with 2D materials in optical waveguides [109,151]. The geometry of a waveguide-based graphene/silicon heterostructure photodetector is shown in Figure 12a. The device can be considered a transistor with zero gate voltage [152]. When a bias voltage of 1.5 V is applied to the two Au electrodes, the responsivity of up to 0.13 A/W can be achieved for 2.75 μm infrared light at room temperature (Figure 12e). The high responsivity in the mid-infrared (MIR) band may be due to the strong absorption of the evanescent light propagating parallel to the graphene layer in the in-plane optical waveguide. Gan et al. reported a similar device that can operate at high frequencies exceeding 20 GHz with a responsivity higher than 0.1 A/W [153], as shown in Figure 12b,f. Ilya et al. further studied the graphene/silicon waveguide Schottky photodiode [154]. The results showed that the high response could be attributed to the extension of the interaction length in the integrated waveguide structure or the plasma of the gold electrode on the waveguide. The effect is shown in Figure 12c,g. This on-chip integrated metal graphene-silicon plasma Schottky photodetector exhibits a responsivity of 85 mA/W at a wavelength of 1.55 μm and reverses bias of 1 V. Due to the low dark current, the noise equivalent power of this device is about 1.1 × 10^−12^ W/Hz^1/2^, which is similar to the most advanced Si Schottky photodetector. Similar to graphene, BP can also be integrated with a waveguide. A miniaturized BP waveguide photodetector is a challenge to maintaining a high responsivity due to the shortening of the optical–matter interaction length. To solve this problem, Ma et al. proposed a method to exploit the slow light effect in photonic crystal waveguides and experimentally verified that this detector could achieve a responsivity of 11.31 A/W at a 0.5 V bias [110]. However, the stability of black phosphorus in the air is relatively low, which limits its wide application. In addition to graphene and BP, detectors based on silicon waveguide integrated MoTe_2_ have also been extensively studied. Maiti et al. propose a strain-engineered photodetector that combines multilayer crystalline MoTe_2_ on a silicon micro loop resonator (Figure 12d) [111]. These MoTe_2_ detectors operate with high responsivity at 1550 nm due to the strain-engineered reduced bandgap and enhanced absorption of microring resonator (MRR) photon lifetime proportional to the fineness of the cavity (Figure 12h).

**Figure 13:**
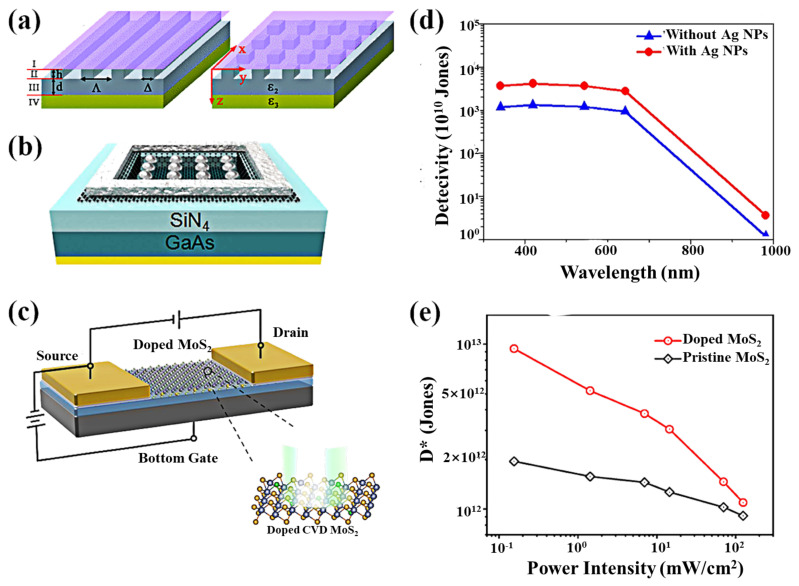
The 2D materials-based plasma photodetectors. (**a**) Schematic view of a graphene monolayer supported on a 1D and 2D SWDG with thickness h, period, and strip/cylinder size. (**b**) Schematic diagram of graphene/GaAs photodetector with AgNPs. (**c**) Schematic diagram of the structure for the doping CVD MoS_2_ photodetector. (**d**) Detectivity of the graphene/GaAs photodetector with and without 100 nm AgNPs under zero bias voltage at five excitation wavelengths. (**e**) Power intensity dependence of D* for the two photodetectors. (**a**) Reproduced with permission from [161]. Copyright American Physical Society, 2012. (**b**,**d**) Reproduced with permission from [113]. Copyright Elsevier, 2018. (**c**,**e**) Reproduced with permission from [106]. Copyright American Chemical Society, 2019.

Recently, the use of field enhancement caused by surface plasmonic excitations to increase the optical response has received great attention for its potential applications in optoelectronics [114,155,156]. Local plasmons in metal nanostructures are first used in combination with graphene to achieve surface-enhanced Raman scattering [157]. The 2D materials-based plasma photodetectors lead to a significant improvement in performance by placing the plasma nanostructures near the contact point [158,159]. The nanostructures with wavelength-specific geometric resonances can be used for selective amplification, potentially allowing filtering and detection [112,160]. Compared to conventional devices, the frequency performance of the device is improved because the plasma structure contributes little to the capacitance, but can significantly reduce the contact resistance. The plasma excitations formed by a monolayer of graphene on sub-wavelength dielectric gratings (SWDGs) via the scattering matrix method reported by Zhan et al. are predicted to have a peak absorbance of 92% for 1D SWDGs and 91% for 2D SWDGs at normal incidence (Figure 13a), which is much higher than the intrinsic absorption of the monolayer of graphene [161]. Thus, the introduction of SWDGs provides an excellent method to enhance the optical absorption of graphene and improve the sensitivity of graphene-based photodetectors. The AgNPs/graphene/GaAs heterostructure (Figure 13b) designed by Lu et al. in 2018, has enhanced responsivity and detectivity across the entire spectral range (Figure 13d) [113]. The surface plasmon resonance (SPR)-enhanced light–matter interaction results in the overlap of the depletion, light-absorption, and surface plasma-enhanced regions beneath the surface of silver nanoparticles. As light is absorbed in the region close to the GaAs surface, the unexpected bulk recombination is suppressed during the drift of the light-generating holes, and thus the photocurrent is increased. Since the electrons in the conduction band of the indirect bandgap semiconductor transit from the valence band to the conduction band, there is a relaxation process to reach the bottom of the conduction band, so part of the energy is wasted in the form of phonons in this process. Therefore, the use of the surface plasmon enhancement effect can provide a greater advantage for the direct bandgap semiconductor heterostructures with a higher light absorption coefficient. Recently, Li et al. improved the optical response of a molybdenum disulfide photodetector using a novel chemical in situ doping of gold chloride hydrates (Figure 13c) [106]. The detector achieves a photoresponsivity of 99.9 A/W at V_DS_ = 0.1 V and V_GS_ = 0 V (Figure 13e). This doping strategy opens up an avenue for the wide application of high-performance photodetectors and offers the possibility of enhancing the optical response of other two-dimensional materials [162,163,164].

## 5. Application Demonstration Based on Photodetectors

**Figure 14:**
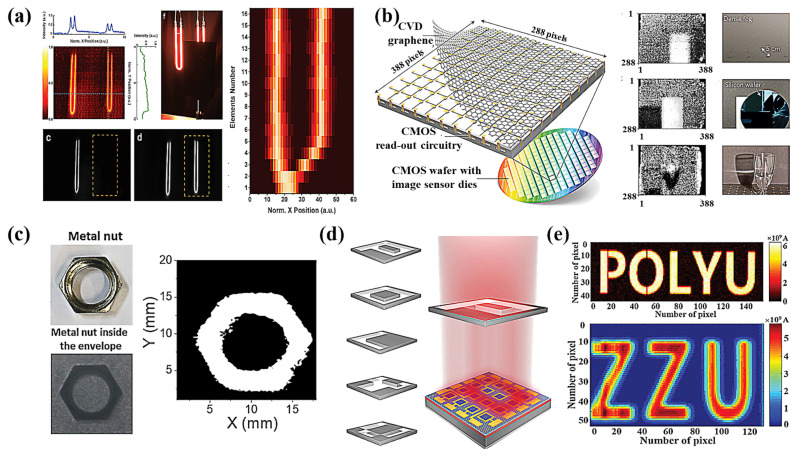
(**a**) Image realized from an advanced dual-band vertical heterostructure GaSe/GaSb linear array photodetector. (**b**) Left: image sensor array based on graphene-complementary metal-oxide–semiconductors (CMOS) integration; right: near-infrared (NIR) and short-wave infrared (SWIR) image of a rectangular block covered in fog, behind a silicon wafer, and a glass of water. (**c**) 2D scanning imaging of the concealed metallic nut in an envelope. (**d**) Left: schematic illustration of the experimental setup for the integrated device to record infrared light imaging sensing. (**e**) The resulting images of “POLYU” and “ZZU” under 4.55 and 10.6 µm illumination, respectively. (**a**) Reproduced with permission from [165]. Copyright Wiley, 2017. (**b**) Reproduced with permission from [166]. Copyright Nature Publishing Group, 2017. (**c**) Reproduced with permission from [102]. Copyright Wiley, 2019. (**d**,**e**) Reproduced with permission from [41]. Copyright Wiley, 2020.

High-performance (ultra-fast light response, broad-spectrum detection, and ultrasensitive) photodetectors play an important role in many areas of daily life. High-sensitivity photoelectric detectors based on 2D materials have an important status in civilian and military applications, with non-contact, non-destructive, long-range, anti-interference capability, and small environmental impact, fast detection speed, and high measurement accuracy being the main directions of today’s photoelectric detection technology. To date, photoelectric detectors have been developed to operate at room temperature for broad-spectrum detection. The excellent performance of photoelectric detectors based on two-dimensional materials can already be compared with the traditional Si and Ge detectors. Therefore, it is appropriate to develop two-dimensional material-based photodetection that is superior to the existing technology. Several difficulties limit the development of 2D photodetectors in industrial applications. One of the greatest difficulties is the enormous area growth of 2D materials, which limits the development of 2D-based focal plane arrays. Hu et al. demonstrated a high-performance two-band photodetector for two-color imaging based on wafer-level 2D GaSe/GaSb vdW vertical heterostructures grown by molecular beam epitaxy (Figure 14a) [165]. The vertical heterostructure is grown in a layer-by-layer epitaxial mode, with wafer-scale multilayer GaSe deposited directly on the n-type GaSb substrate. Indium-tin oxide (ITO) and indium (In) as electrodes are formed on the surface of GaSe and GaSb as anode and cathode, respectively. Indium tin oxide (ITO) was chosen for the anode electrode placed on top of GaSe because this material makes good ohmic contact with GaSe and easily passes light to improve energy absorption within GaSe. By introducing a strong local electric field, dark currents can be suppressed and the efficiency of photogenerated carrier separation can be improved so that a device with broadband optical response has excellent dual-band detection capability. This is the first time a 2D material-based photodetector has been used to demonstrate practical broadband applications from visible to short wavelengths. This heterogeneous photodiode structure has both the excellent optical responsivity of 2D GaSe in the visible range and the excellent light detection in the infrared region of conventional GaSb, thus providing a viable method of using 2D materials at room temperature. Subsequently, the integration potential was shown through the implementation of an image sensor using an array of graphene-quantum-dot photodetectors that can be used as an image sensor with high sensitivity to both visible and short-wave infrared light (Figure 14b) [166]. The device was fabricated by transferring graphene grown by chemical vapor deposition onto a silicon-based substrate with integrated readout circuitry and then conforming the graphene to define each pixel. Finally, PbS quantum dots were deposited onto the graphene layer by spin-coating. The high-resolution imaging of the graphene quantum dot CMOS sensor in the visible and short-wavelength bands reveal an effective method for achieving monolithic integration of 2D material sensors, demonstrating its feasibility of layer-by-layer stacking of 2D materials in optoelectronic circuits. More excitingly, Xu et al. designed a two-dimensional grating scanning imaging experiment based on a semimetallic photodetector (Figure 14c) [102]. A metal nut inside an envelope was placed on the focal plane to mimic the detection of a hidden object. The resulting 80 × 80-pixel image clearly shows the shape of the metal nut, demonstrating the rapid imaging application of our device. Jie et al. developed two methods to check the infrared imaging capabilities of photodetectors (Figure 14d,e) [41]. First, with the help of a mask made in the laboratory controlled by the movement of the 2D turntable, the position-resolved photocurrent can be recorded by a computer programmed by software, thereby generating an image with a resolution of 4.55um. On the other hand, an array of 4 × 4 devices was fabricated to evaluate its potential application in an integrated light detection system. When illuminating at mid-infrared wavelengths, photocurrents are recorded from pixels under the exposed region, while dark currents are collected from pixels under the shaded region, producing the corresponding letter shapes. These results clearly confirm that PtTe_2_/Si photodetectors have great potential for NIR-MIR imaging applications and that PtTe_2_/Si photodetectors can operate well at room temperature, which will help address existing technical bottlenecks for future scientific and industrial applications.

Due to their tremendous advantages, 2D materials have already shown great potential in PV solar cells [167]. With the trend toward thinner and more portable PV devices, atomically thin 2D materials have become the obvious choice for integration into the next generation of PV technology. In PV solar cells, electrons and holes are generated as the material absorbs light, and the electric field formed by the barrier at the interface between the different materials efficiently separates the electrons and holes. Heterojunctions dominate conventional solar cells. Excess electrons diffuse from n-type to p-type, and excess holes diffuse from p-type to n-type, leading to a depletion zone, which creates an electric field at the junction. The electric field separates the electron–hole pairs generated in the depletion zone of the heterojunction due to photoexcitation. From the point of view of light absorption, it is necessary to increase the thickness of PV cells to increase the efficiency of light absorption. However, the performance of solar cells based on 2D materials seems to exceed that of conventional PV cells. Since the bandgap of TMD materials is distributed in the visible near-infrared band, this gives them great potential for PV cell applications. In addition, most monolayer TMDs are direct bandgap semiconductors, which have high radiative efficiency. Combined with their higher absorption coefficient per unit thickness, they have a massive advantage in ultra-thin PV devices with high absorption. A 2D bilayer WSe_2_/MoS_2_ p–n lateral heterojunction solar cell designed by Tsai et al. exhibited a power conversion efficiency of up to 2.56% under AM 1.5G [168]. Due to this lateral heterostructure, the entire depletion region is exposed to light, so it has excellent omnidirectional light collection characteristics. Wong et al. prepared an ultra-thin WSe_2_/MoS_2_ vdW heterostructure [169]. The EQE exceeds 50%, and the light absorption rate in the experiment can reach 90%. Since the heterostructures can be produced at a low cost and over a large area and can be fully compatible with various flexible and transparent substrates, this fully illustrates the potential applications of PV devices based on 2D materials.

## 6. Conclusions and Outlook

In this paper, we present the latest advances in the latest photodetectors based on 2D materials. Photodetection based on 2D materials shows a bright future and attractive applications due to the many outstanding performance characteristics currently available. Due to the extremely thin thickness of 2D materials, light absorption may be insufficient, limiting EQE and detection capability [170]. Although many 2D photodetectors exhibit high optical responsivity, much of this is attributed to extended carrier lifetimes at the expense of response speed. Photodetectors with high optical responsivity can be used for low-light detection. However, the high gain due to extended carrier lifetimes also leads to high noise and fails to improve the detectivity. Moreover, for future detector arrays, the high gain will require high material uniformity and process technology requirements, making the products and applications more difficult [171]. Graphene and certain 2D materials show high bandwidths, especially when integrated with waveguides. However, the optical responsivity is low, and the dark current suppression is still required for practical applications. Dark currents and other noise sources can be suppressed by introducing p–n junctions or Schottky barriers. Therefore, there is a strong need for 2D photodetectors with good optical responsivity, wide bandwidth, and high detectivity. The optical response of photodetectors is typically low when measuring devices at moderate source-drain and gate voltages. It can also be increased by increasing the V_DS_, but such ultra-high voltages would consume significant amounts of power and would be impractical for photodetection applications. Unfortunately, enhanced photocurrent methods have their drawbacks, such as the lack of stability of quantum dots, weak enhancement of surface plasmon resonances, and complicated preparation of heterojunctions [172]. For TMDs, direct bandgap properties capable of achieving high optical absorption efficiencies occur only in monolayer structures. Additionally, the electrical and optoelectronic properties of atomically thin 2D layered materials are susceptible to environmental conditions. Some 2D materials, especially narrow bandgap materials, are unstable in ambient air [173]. Another major challenge is the high dark current of photoconductive 2D photodetectors, resulting in lower specific detectivity. Finally, one of the main challenges for 2D materials is the large-scale material deposition of high-quality thin films with high reliability and low variability.

Photodetectors with graphene-based FETs have triggered a great deal of research into semimetal-based photodetectors [44]. Because of the gapless electronic structures of semimetal endow them with broadband photoresponses down to the far-infrared spectral region [174]. Furthermore, in the absence of a bandgap, the short life of photoexcited carriers is increased by fast electron–electron scattering, thus increasing the operating speed of the semimetals. Many approaches, such as plasma technology, integration of microcavities, and optical waveguides, have proven to improve device performance [175,176]. Considering the practical application of the device, further improvement of the device performance in terms of uniformity and stability is required. The prospects for the commercialization of photodetectors will depend not only on the performance of the detectors but also on some of their unique advantages and capabilities and the ability to enable large-scale, low-cost, high-quality products, and to establish large-scale integration with existing photonic and electronic platforms [177]. Most current high-performance photodetectors are based on thin layers or heterostructures obtained by mechanical exfoliation. Since the heterostructure transfer process is complex and challenging to use in practical applications, chemical vapor deposition (CVD) growth of large area, high-quality layered materials is critical for practical applications. Photodetectors based on topological Weyl semimetals offer many other advantages over conventional photodetectors, including broadband light response from UV to IR, and mechanical flexibility [178]. In addition, the topological effect may also provide control mechanisms for specific quantum degrees of freedom, such as the cyclic selection rules associated with the chiral nature of the Weyl cone, which help to distinguish the helicity of light, resulting in helicity-sensitive photodetectors based on Weyl semimetallic and chiral fermionic materials. Many research groups have realized powerful and flexible Weyl semimetallic photodetectors. This implies that the focus on topology enhances the shift current response of Weyl semimetals. Addressing the difficulties faced in traditional photoelectric detection through topology may be an important way to implement the physics of topology for practical applications.

## Figures and Tables

**Table 1:** Performance of 2D and bulk material phototransistors.

Enhanced Type	Active Materials	Mechanism	Spectral Range	Responsivity	Detectivity (Jones)	Response Time	Ref.
Metal/2D	MoS_2_	PB	980, 1550 nm	1.9 × 10^4^ A/W			[85]
	MoS2	PG	550 nm	10^5^ A/W	10^14^		[86]
	Perovskite/Au	PC	405 nm	1.6 × 10^7^ A/W		81 μs	[87]
	PdSe_2_	PG	1.06 um	708 A/W	1.31 × 10^9^	220 ms	[88]
	MoSe2	PC	670–1458 nm	10.1 A/W			[89]
	WSe2	PV	532 nm	2.31 A/W	9.16 × 10^11^		[90]
	TaIrTe4	TP	4 um	130.2 mA/W			[83]
	T_d_-WTe_2_	TP	450–2400 nm				[91]
	T_d_-MoTe_2_	PTE	532 nm–10.6 μm	0.40 mA/W	1.07 × 10^8^	43 μs	[74]
Heterostructure	MoS_2_	PG	637 nm	96.8 A/W	4.75 × 10^14^	400 μs	[92]
	p-MoS2/n- MoS2	PG	640–800 nm	7 × 10^4^ A/W	3.5 × 10^14^	10 ms	[93]
	Graphene/MoS2	PV	450–700 nm	1.1 × 10^5^ A/W	1.4 × 10^14^	100 ms	[94]
	MoS_2_/WS_2_	PV	532 nm	4.36 mA/W	4.36 × 10^13^	4 ms	[95]
	MoS_2_/Si	PV	350–1100 nm	908.2 mA/W	1.889 × 10^13^	56 ns	[96]
	WSe_2_/MoS_2_	PV	450–800 nm	2700 A/W	5 × 10^11^	17 ms	[47]
	Perovskite/CdS	PV	350–750 nm	0.48 AW	2.1 × 10^13^	0.54 ms	[97]
	PbI_2_/graphene/PET	PV	480 nm	45 A/W		35 μs	[98]
	SnS_2_/MoO_3_	PV	515 nm	2.3 × 10^3^ A/W	3.2 × 10^12^	2.72 ms	[99]
	Graphene/GaAs	PV	650 nm	1321 A/W	3.24 × 10^8^	119 ms	[100]
	Gr_1_/ Perovskite/ Gr_2_	PV	457 nm	3 × 10^9^ A/W	8.7 × 10^1^	50 μs	[101]
	PtTe2/Si	PV	200 nm–10.6 µm		6.92 × 10^9^	2.4 μs	[41]
	PtTe2/graphene	PV	2600 um	1.6 A/W		20 μs	[102]
	Gr_1_/BP/Gr_2_	PV	0.5–3.5 um	1.43 A/W	8.67 × 10^8^	1.8 ns	[103]
Hybrid structure	Monolayer MoS_2_	PG	532 nm	430 A/W	10^11^	72 ms	[104]
	graphene/Cu_2_O	PG	450 nm	10^10^ A/W	1.4 × 10^12^	273 ms	[39]
	InP/BP	PG	405 nm	10^9^ A/W	4.5 × 10^16^	5 ms	[42]
	MoS2/ZnCdSe QD	PV	<700 nm	3.7 × 10^4^ A/W	10^12^	0.3 s	[105]
	Ti_2_O_3_/ Graphene	PC	4.5–10 um	300 A/W	7 × 10^8^	1.2 ms	[53]
	Chemical doping MoS_2_	PG	450–750 nm	99.9 A/W	9.4 × 10^12^	16.6 s	[106]
Cavities	graphene	PV, PTE	96 um	0.23 A/W	2.8 × 10^10^	265 ns	[107]
	Gr/TiO_2_	PG	325 nm	475.5 A/W		220 ms	[108]
Waveguides	MoTe_2_/graphene	PG	1300 nm	0.2 A/W		19 ps	[109]
	BP	PB	3.725, 3.775, 3.825 um	11.31 A/W		0.3 ms	[110]
	graphene	PB, PC	2 um	70 mA/W			[73]
	MoTe_2_	PV	1500 nm	0.5 A W		3.2 ns	[111]
Plasmonics	graphene	PTE	500–900 nm	0.125 mA/W		0.4 ps	[40]
	graphene	PV	2400 nm	0.12 A/W			[112]
	graphene/GaAs/Ag NPs	PV	325–980 nm	210 mA/W	2.98 × 10^13^		[113]
	Au NPs/WS_2_/MoS_2_	PV	532 nm	0.49 A/W			[114]
Bulk detector	Si	PC	630nm	1.5 A/W		0.11 s	[115]
	Ge	PC	1.7 um	0.6 A/W	10^11^	0.87 μs	[25]
	Ge	PC	390 nm	0.63 A/W	7 × 10^11^		[116]
	SiC	PC	445 nm	12.2 A/W	1.13 × 10^9^	0.39 s	[117]
	GaN/Sn:Ga_2_O_3_	PV	254 nm	3.05	1.69 × 10^13^	18 ms	[118]

## Data Availability

The data presented in this study are available on request from the corresponding authors.

## References

[1] Fang H., Wu P., Wang P., Zheng Z., Tang Y.J., Ho C., Chen G., Wang Y., Shan C., Cheng X. (2019). Global Photocurrent Generation in Phototransistors Based on Single-Walled Carbon Nanotubes toward Highly Sensitive Infrared Detection. Adv. Opt. Mater..

[2] Han L., Bai L., Dong S.J. (2014). Self-powered visual ultraviolet photodetector with Prussian blue electrochromic display. Chem. Commun..

[3] Shehzad K., Xu Y. (2020). Graphene light-field camera. Nat. Photonics.

[4] Formisano V., Atreya S., Encrenaz T., Ignatiev N., Giuranna M. (2004). Detection of methane in the atmosphere of Mars. Science.

[5] Mueller T., Xia F., Avouris P. (2010). Graphene photodetectors for high-speed optical communications. Nat. Photonics.

[6] Kim S., Lim Y.T., Soltesz E.G., De Grand A.M., Lee J., Nakayama A., Parker J.A., Mihaljevic T., Laurence R.G., Dor D.M. (2004). Near-infrared fluorescent type II quantum dots for sentinel lymph node mapping. Nat. Biotechnol..

[7] Xiao P., Mao J., Ding K., Luo W., Hu W., Zhang X., Zhang X., Jie J. (2018). Solution-Processed 3D RGO-MoS2 /Pyramid Si Heterojunction for Ultrahigh Detectivity and Ultra-Broadband Photodetection. Adv. Mater..

[8] Cai X.K., Luo Y.T., Liu B., Cheng H.M. (2018). Preparation of 2D material dispersions and their applications. Chem. Soc. Rev..

[9] Geim A.K., Grigorieva I.V. (2013). Van der Waals heterostructures. Nature.

[10] Zong X., Hu H., Ouyang G., Wang J., Shi R., Zhang L., Zeng Q., Zhu C., Chen S., Cheng C. (2020). Black phosphorus-based van der Waals heterostructures for mid-infrared light-emission applications. Light Sci. Appl..

[11] Mak K.F., He K.L., Lee C., Lee G.H., Hone J., Heinz T.F., Shan J. (2013). Tightly bound trions in monolayer MoS2. Nat. Mater..

[12] Mak K.F., He K.L., Shan J., Heinz T.F. (2012). Control of valley polarization in monolayer MoS2 by optical helicity. Nat. Nanotechnol..

[13] Xia F., Wang H., Xiao D., Dubey M., Ramasubramaniam A. (2014). Two-dimensional material nanophotonics. Nat. Photonics.

[14] Kim C.O., Kim S., Shin D.H., Kang S.S., Kim J.M., Jang C.W., Joo S.S., Lee J.S., Kim J.H., Choi S.H. (2014). High photoresponsivity in an all-graphene p-n vertical junction photodetector. Nat. Commun..

[15] Zhang Y.Z., Liu T., Meng B., Li X.H., Liang G.Z., Hu X.N., Wang Q.J. (2013). Broadband high photoresponse from pure monolayer graphene photodetector. Nat. Commun..

[16] Wu J., Koon G.K.W., Xiang D., Han C., Toh C.T., Kulkarni E.S., Verzhbitskiy I., Carvalho A., Rodin A.S., Koenig S.P. (2015). Colossal Ultraviolet Photoresponsivity of Few-Layer Black Phosphorus. ACS Nano.

[17] Kufer D., Konstantatos G. (2015). Highly Sensitive, Encapsulated MoS2 Photodetector with Gate Controllable Gain and Speed. Nano Lett..

[18] Long M., Wang P., Fang H., Hu W. (2018). Progress, Challenges, and Opportunities for 2D Material Based Photodetectors. Adv. Funct. Mater..

[19] Konstantatos G., Badioli M., Gaudreau L., Osmond J., Bernechea M., Garcia de Arquer F.P., Gatti F., Koppens F.H. (2012). Hybrid graphene-quantum dot phototransistors with ultrahigh gain. Nat. Nanotechnol..

[20] Yang Y., Wang X., Wang C., Song Y., Zhang M., Xue Z., Wang S., Zhu Z., Liu G., Li P. (2020). Ferroelectric Enhanced Performance of a GeSn/Ge Dual-Nanowire Photodetector. Nano Lett..

[21] Wu G., Wang X., Chen Y., Wu S., Wu B., Jiang Y., Shen H., Lin T., Liu Q., Wang X. (2020). MoTe2 p-n Homojunctions Defined by Ferroelectric Polarization. Adv. Mater..

[22] Wang X., Wang P., Wang J., Hu W., Zhou X., Guo N., Huang H., Sun S., Shen H., Lin T. (2015). Ultrasensitive and Broadband MoS(2) Photodetector Driven by Ferroelectrics. Adv. Mater..

[23] Jiang W., Zheng T., Wu B., Jiao H., Wang X., Chen Y., Zhang X., Peng M., Wang H., Lin T. (2020). A versatile photodetector assisted by photovoltaic and bolometric effects. Light Sci. Appl..

[24] Chen X., Wang D., Wang T., Yang Z., Zou X., Wang P., Luo W., Li Q., Liao L., Hu W. (2019). Enhanced Photoresponsivity of a GaAs Nanowire Metal-Semiconductor-Metal Photodetector by Adjusting the Fermi Level. ACS Appl. Mater. Interfaces.

[25] Huo N., Konstantatos G. (2018). Recent Progress and Future Prospects of 2D-Based Photodetectors. Adv. Mater..

[26] Nair R.R., Blake P., Grigorenko A.N., Novoselov K.S., Booth T.J., Stauber T., Peres N.M.R., Geim A.K. (2008). Fine structure constant defines visual transparency of graphene. Science.

[27] Mak K.F., Lee C., Hone J., Shan J., Heinz T.F. (2010). Atomically Thin MoS2: A New Direct-Gap Semiconductor. Phys. Rev. Lett..

[28] Tsai D.S., Liu K.K., Lien D.H., Tsai M.L., Kang C.F., Lin C.A., Li L.J., He J.H. (2013). Few-Layer MoS2 with High Broadband Photogain and Fast Optical Switching for Use in Harsh Environments. ACS Nano.

[29] Chitara B., Panchakarla L.S., Krupanidhi S.B., Rao C.N. (2011). Infrared photodetectors based on reduced graphene oxide and graphene nanoribbons. Adv. Mater..

[30] Youngblood N., Chen C., Koester S.J., Li M. (2015). Waveguide-integrated black phosphorus photodetector with high responsivity and low dark current. Nat. Photonics.

[31] Ryzhii V., Ryzhii M., Ryabova N., Mitin V., Otsuji T. (2009). Graphene Nanoribbon Phototransistor: Proposal and Analysis. Jpn. J. Appl. Phys..

[32] Gao L., Chen C., Zeng K., Ge C., Yang D., Song H., Tang J. (2016). Broadband, sensitive and spectrally distinctive SnS2 nanosheet/PbS colloidal quantum dot hybrid photodetector. Light Sci. Appl..

[33] Ling Z.P., Yang R., Chai J.W., Wang S.J., Leong W.S., Tong Y., Lei D., Zhou Q., Gong X., Chi D.Z. (2015). Large-scale two-dimensional MoS(2) photodetectors by magnetron sputtering. Opt. Express.

[34] Radisavljevic B., Radenovic A., Brivio J., Giacometti V., Kis A. (2011). Single-layer MoS2 transistors. Nat. Nanotechnol..

[35] Lopez-Sanchez O., Lembke D., Kayci M., Radenovic A., Kis A. (2013). Ultrasensitive photodetectors based on monolayer MoS2. Nat. Nanotechnol..

[36] Konstantatos G. (2018). Current status and technological prospect of photodetectors based on two-dimensional materials. Nat. Commun..

[37] Fang Y., Armin A., Meredith P., Huang J. (2018). Accurate characterization of next-generation thin-film photodetectors. Nat. Photonics.

[38] García de Arquer F.P., Armin A., Meredith P., Sargent E.H. (2017). Solution-processed semiconductors for next-generation photodetectors. Nat. Rev. Mater..

[39] Liu Q., Tian H., Li J., Hu A., He X., Sui M., Guo X. (2019). Hybrid Graphene/Cu2O Quantum Dot Photodetectors with Ultrahigh Responsivity. Adv. Opt. Mater..

[40] Shautsova V., Sidiropoulos T., Xiao X., Gusken N.A., Black N.C.G., Gilbertson A.M., Giannini V., Maier S.A., Cohen L.F., Oulton R.F. (2018). Plasmon induced thermoelectric effect in graphene. Nat. Commun..

[41] Zeng L., Wu D., Jie J., Ren X., Hu X., Lau S.P., Chai Y., Tsang Y.H. (2020). Van der Waals Epitaxial Growth of Mosaic-Like 2D Platinum Ditelluride Layers for Room-Temperature Mid-Infrared Photodetection up to 10.6 microm. Adv. Mater..

[42] Kwak D.H., Ramasamy P., Lee Y.S., Jeong M.H., Lee J.S. (2019). High-Performance Hybrid InP QDs/Black Phosphorus Photodetector. ACS Appl. Mater. Interfaces.

[43] Mueller T., Xia F., Freitag M., Tsang J., Avouris P. (2009). Role of contacts in graphene transistors: A scanning photocurrent study. Phys. Rev. B.

[44] Xia F.N., Mueller T., Golizadeh-Mojarad R., Freitag M., Lin Y.M., Tsang J., Perebeinos V., Avouris P. (2009). Photocurrent Imaging and Efficient Photon Detection in a Graphene Transistor. Nano Lett..

[45] Peters E.C., Lee E.J.H., Burghard M., Kern K. (2010). Gate dependent photocurrents at a graphene p-n junction. Appl. Phys. Lett..

[46] Rao G., Freitag M., Chiu H.Y., Sundaram R.S., Avouris P. (2011). Raman and Photocurrent Imaging of Electrical Stress-Induced p-n Junctions in Graphene. ACS Nano.

[47] Shin G.H., Park C., Lee K.J., Jin H.J., Choi S.Y. (2020). Ultrasensitive Phototransistor Based on WSe2-MoS2 van der Waals Heterojunction. Nano Lett..

[48] Xu X., Gabor N.M., Alden J.S., van der Zande A.M., McEuen P.L. (2010). Photo-thermoelectric effect at a graphene interface junction. Nano Lett..

[49] Yu X., Li Y., Hu X., Zhang D., Tao Y., Liu Z., He Y., Haque M.A., Liu Z., Wu T. (2018). Narrow bandgap oxide nanoparticles coupled with graphene for high performance mid-infrared photodetection. Nat. Commun..

[50] Sun Z., Liu Z., Li J., Tai G.A., Lau S.P., Yan F. (2012). Infrared photodetectors based on CVD-grown graphene and PbS quantum dots with ultrahigh responsivity. Adv. Mater..

[51] Li J., Niu L., Zheng Z., Yan F. (2014). Photosensitive graphene transistors. Adv. Mater..

[52] Fang H., Hu W. (2017). Photogating in Low Dimensional Photodetectors. Adv. Sci..

[53] Farmer D.B., Golizadeh-Mojarad R., Perebeinos V., Lin Y.M., Tulevski G.S., Tsang J.C., Avouris P. (2009). Chemical doping and electron-hole conduction asymmetry in graphene devices. Nano Lett..

[54] Lee E.J., Balasubramanian K., Weitz R.T., Burghard M., Kern K. (2008). Contact and edge effects in graphene devices. Nat. Nanotechnol..

[55] Park J., Ahn Y.H., Ruiz-Vargas C. (2009). Imaging of photocurrent generation and collection in single-layer graphene. Nano Lett..

[56] Vasilyev Y.B. (2020). On the Origin of Photocurrents in Pristine Graphene. Semiconductors.

[57] Ma Q., Lui C.H., Song J.C.W., Lin Y., Kong J.F., Cao Y., Dinh T.H., Nair N.L., Fang W., Watanabe K. (2019). Giant intrinsic photoresponse in pristine graphene. Nat. Nanotechnol..

[58] Yin J.B., Peng H.L. (2019). Asymmetry allows photocurrent in intrinsic graphene. Nat. Nanotechnol..

[59] Gabor N.M., Song J.C., Ma Q., Nair N.L., Taychatanapat T., Watanabe K., Taniguchi T., Levitov L.S., Jarillo-Herrero P. (2011). Hot carrier-assisted intrinsic photoresponse in graphene. Science.

[60] Lemme M.C., Koppens F.H., Falk A.L., Rudner M.S., Park H., Levitov L.S., Marcus C.M. (2011). Gate-activated photoresponse in a graphene p-n junction. Nano Lett..

[61] Song J.C., Rudner M.S., Marcus C.M., Levitov L.S. (2011). Hot carrier transport and photocurrent response in graphene. Nano Lett..

[62] Sun D., Aivazian G., Jones A.M., Ross J.S., Yao W., Cobden D., Xu X. (2012). Ultrafast hot-carrier-dominated photocurrent in graphene. Nat. Nanotechnol..

[63] Patil V., Capone A., Strauf S., Yang E.H. (2013). Improved photoresponse with enhanced photoelectric contribution in fully suspended graphene photodetectors. Sci Rep..

[64] Lu X.W., Sun L., Jiang P., Bao X.H. (2019). Progress of Photodetectors Based on the Photothermoelectric Effect. Adv. Mater..

[65] Vandermeiren W., Stiens J., Shkerdin G., Kotov V., Tandt C.D., Vounckx R., Duarte F.J. (2010). Laser Pulse Phenomena and Applications.

[66] Freitag M., Low T., Xia F., Avouris P. (2012). Photoconductivity of biased graphene. Nat. Photonics.

[67] Guo J., Li J., Liu C., Yin Y., Wang W., Ni Z., Fu Z., Yu H., Xu Y., Shi Y. (2020). High-performance silicon-graphene hybrid plasmonic waveguide photodetectors beyond 1.55 mum. Light Sci. Appl..

[68] Mahjoub A.M., Suzuki S., Ouchi T., Aoki N., Miyamoto K., Yamaguchi T., Omatsu T., Ishibashi K., Ochiai Y. (2015). Terahertz bolometric detection by thermal noise in graphene field effect transistor. Appl. Phys. Lett..

[69] Zak A., Andersson M.A., Bauer M., Matukas J., Lisauskas A., Roskos H.G., Stake J. (2014). Antenna-integrated 0.6 THz FET direct detectors based on CVD graphene. Nano Lett..

[70] Muraviev A.V., Rumyantsev S.L., Liu G., Balandin A.A., Knap W., Shur M.S. (2013). Plasmonic and bolometric terahertz detection by graphene field-effect transistor. Appl. Phys. Lett..

[71] Kim M.H., Yan J., Suess R.J., Murphy T.E., Fuhrer M.S., Drew H.D. (2013). Photothermal response in dual-gated bilayer graphene. Phys. Rev. Lett..

[72] Yan J., Kim M.H., Elle J.A., Sushkov A.B., Jenkins G.S., Milchberg H.M., Fuhrer M.S., Drew H.D. (2012). Dual-gated bilayer graphene hot-electron bolometer. Nat. Nanotechnol..

[73] Vora H., Kumaravadivel P., Nielsen B., Du X. (2012). Bolometric response in graphene based superconducting tunnel junctions. Appl. Phys. Lett..

[74] Li H., He H., Lu H.Z., Zhang H., Liu H., Ma R., Fan Z., Shen S.Q., Wang J. (2016). Negative magnetoresistance in Dirac semimetal Cd3As2. Nat. Commun..

[75] Ma J., Gu Q., Liu Y., Lai J., Yu P., Zhuo X., Liu Z., Chen J.H., Feng J., Sun D. (2019). Nonlinear photoresponse of type-II Weyl semimetals. Nat. Mater..

[76] Guo C., Hu Y.B., Chen G., Wei D.C., Zhang L.B., Chen Z.Q.Z., Guo W.L., Xu H., Kuo C.N., Lue C.S. (2020). Anisotropic ultrasensitive PdTe2-based phototransistor for room-temperature long-wavelength detection. Sci. Adv..

[77] Osterhoudt G.B., Diebel L.K., Gray M.J., Yang X., Stanco J., Huang X., Shen B., Ni N., Moll P.J.W., Ran Y. (2019). Colossal mid-infrared bulk photovoltaic effect in a type-I Weyl semimetal. Nat. Mater..

[78] Weng H., Fang C., Fang Z., Dai X. (2017). A new member of the topological semimetals family. Natl. Sci. Rev..

[79] Yu W., Li S., Zhang Y., Ma W., Sun T., Yuan J., Fu K., Bao Q. (2017). Near-Infrared Photodetectors Based on MoTe2/Graphene Heterostructure with High Responsivity and Flexibility. Small.

[80] Hou W., Azizimanesh A., Sewaket A., Pena T., Watson C., Liu M., Askari H., Wu S.M. (2019). Strain-based room-temperature non-volatile MoTe2 ferroelectric phase change transistor. Nat. Nanotechnol..

[81] Lu G., Yu K., Wen Z., Chen J. (2013). Semiconducting graphene: Converting graphene from semimetal to semiconductor. Nanoscale.

[82] Liu J., Xia F., Xiao D., Garcia de Abajo F.J., Sun D. (2020). Semimetals for high-performance photodetection. Nat. Mater..

[83] Chan C.K., Lindner N.H., Refael G., Lee P.A. (2017). Photocurrents in Weyl semimetals. Phys. Rev. B.

[84] De Sanctis A., Mehew J.D., Craciun M.F., Russo S. (2018). Graphene-Based Light Sensing: Fabrication, Characterisation, Physical Properties and Performance. Materials.

[85] Wu J.Y., Chun Y.T., Li S., Zhang T., Wang J., Shrestha P.K., Chu D. (2018). Broadband MoS2 Field-Effect Phototransistors: Ultrasensitive Visible-Light Photoresponse and Negative Infrared Photoresponse. Adv. Mater..

[86] Liao F., Deng J., Chen X., Wang Y., Zhang X., Liu J., Zhu H., Chen L., Sun Q., Hu W. (2020). A Dual-Gate MoS2 Photodetector Based on Interface Coupling Effect. Small.

[87] Yang Z., Deng Y., Zhang X., Wang S., Chen H., Yang S., Khurgin J., Fang N.X., Zhang X., Ma R. (2018). High-Performance Single-Crystalline Perovskite Thin-Film Photodetector. Adv. Mater..

[88] Liang Q., Wang Q., Zhang Q., Wei J., Lim S.X., Zhu R., Hu J., Wei W., Lee C., Sow C. (2019). High-Performance, Room Temperature, Ultra-Broadband Photodetectors Based on Air-Stable PdSe2. Adv. Mater..

[89] Kim S., Maassen J., Lee J., Kim S.M., Han G., Kwon J., Hong S., Park J., Liu N., Park Y.C. (2018). Interstitial Mo-Assisted Photovoltaic Effect in Multilayer MoSe2 Phototransistors. Adv. Mater..

[90] Zhou C., Raju S., Li B., Chan M., Chai Y., Yang C.Y. (2018). Self-Driven Metal-Semiconductor-Metal WSe2 Photodetector with Asymmetric Contact Geometries. Adv. Funct. Mater..

[91] Wang Q., Zheng J., He Y., Cao J., Liu X., Wang M., Ma J., Lai J., Lu H., Jia S. (2019). Robust edge photocurrent response on layered type II Weyl semimetal WTe2. Nat. Commun..

[92] Tu L., Cao R., Wang X., Chen Y., Wu S., Wang F., Wang Z., Shen H., Lin T., Zhou P. (2020). Ultrasensitive negative capacitance phototransistors. Nat. Commun..

[93] Huo N., Konstantatos G. (2017). Ultrasensitive all-2D MoS2 phototransistors enabled by an out-of-plane MoS2 PN homojunction. Nat. Commun..

[94] Deng W., Chen Y., You C., Liu B., Yang Y., Shen G., Li S., Sun L., Zhang Y., Yan H. (2018). High Detectivity from a Lateral Graphene-MoS2 Schottky Photodetector Grown by Chemical Vapor Deposition. Adv. Electron. Mater..

[95] Wu W., Zhang Q., Zhou X., Li L., Su J., Wang F., Zhai T. (2018). Self-powered photovoltaic photodetector established on lateral monolayer MoS2-WS2 heterostructures. Nano Energy.

[96] Qiao S., Cong R., Liu J., Liang B., Fu G., Yu W., Ren K., Wang S., Pan C. (2018). A vertically layered MoS2/Si heterojunction for an ultrahigh and ultrafast photoresponse photodetector. J. Mater. Chem. C.

[97] Cao F., Meng L., Wang M., Tian W., Li L. (2019). Gradient Energy Band Driven High-Performance Self-Powered Perovskite/CdS Photodetector. Adv. Mater..

[98] Zhang J., Huang Y., Tan Z., Li T., Zhang Y., Jia K., Lin L., Sun L., Chen X., Li Z. (2018). Low-Temperature Heteroepitaxy of 2D PbI2 /Graphene for Large-Area Flexible Photodetectors. Adv. Mater..

[99] Gao J., Yang H., Mao H., Liu T., Zheng Y., Wang Y., Xiang D., Han C., Chen W. (2020). Out-of-Plane Homojunction Enabled High Performance SnS2 Lateral Phototransistor. Adv. Opt. Mater..

[100] Tian H., Hu A., Liu Q., He X., Guo X. (2020). Interface-Induced High Responsivity in Hybrid Graphene/GaAs Photodetector. Adv. Opt. Mater..

[101] Bera K.P., Haider G., Huang Y.T., Roy P.K., Paul Inbaraj C.R., Liao Y.M., Lin H.I., Lu C.H., Shen C., Shih W.Y. (2019). Graphene Sandwich Stable Perovskite Quantum-Dot Light-Emissive Ultrasensitive and Ultrafast Broadband Vertical Phototransistors. ACS Nano.

[102] Xu H., Guo C., Zhang J., Guo W., Kuo C.N., Lue C.S., Hu W., Wang L., Chen G., Politano A. (2019). PtTe2 -Based Type-II Dirac Semimetal and Its van der Waals Heterostructure for Sensitive Room Temperature Terahertz Photodetection. Small.

[103] Chang T.Y., Chen P.L., Yan J.H., Li W.Q., Zhang Y.Y., Luo D.I., Li J.X., Huang K.P., Liu C.H. (2020). Ultra-Broadband, High Speed, and High-Quantum-Efficiency Photodetectors Based on Black Phosphorus. ACS Appl. Mater. Interfaces.

[104] Huang Y., Zhuge F., Hou J., Lv L., Luo P., Zhou N., Gan L., Zhai T. (2018). Van der Waals Coupled Organic Molecules with Monolayer MoS2 for Fast Response Photodetectors with Gate-Tunable Responsivity. ACS Nano.

[105] Zhang S., Wang X., Chen Y., Wu G., Tang Y., Zhu L., Wang H., Jiang W., Sun L., Lin T. (2019). Ultrasensitive Hybrid MoS2-ZnCdSe Quantum Dot Photodetectors with High Gain. ACS Appl. Mater. Interfaces.

[106] Li S., Chen X., Liu F., Chen Y., Liu B., Deng W., An B., Chu F., Zhang G., Li S. (2019). Enhanced Performance of a CVD MoS2 Photodetector by Chemical in Situ n-Type Doping. ACS Appl. Mater. Interfaces.

[107] Deng T., Zhang Z., Liu Y., Wang Y., Su F., Li S., Zhang Y., Li H., Chen H., Zhao Z. (2019). Three-Dimensional Graphene Field-Effect Transistors as High-Performance Photodetectors. Nano Lett..

[108] Li S., Yin W., Li Y., Sun J., Zhu M., Liu Z., Deng T. (2019). High sensitivity ultraviolet detection based on three-dimensional graphene field effect transistors decorated with TiO_2_ NPs. Nanoscale.

[109] Flory N., Ma P., Salamin Y., Emboras A., Taniguchi T., Watanabe K., Leuthold J., Novotny L. (2020). Waveguide-integrated van der Waals heterostructure photodetector at telecom wavelengths with high speed and high responsivity. Nat. Nanotechnol..

[110] Ma Y., Dong B., Wei J., Chang Y., Huang L., Ang K.W., Lee C. (2020). High-Responsivity Mid-Infrared Black Phosphorus Slow Light Waveguide Photodetector. Adv. Opt. Mater..

[111] Maiti R., Patil C., Saadi M.A.S.R., Xie T., Azadani J.G., Uluutku B., Amin R., Briggs A.F., Miscuglio M., Van Thourhout D. (2020). Strain-engineered high-responsivity MoTe2 photodetector for silicon photonic integrated circuits. Nat. Photonics.

[112] Bandurin D.A., Svintsov D., Gayduchenko I., Xu S.G., Principi A., Moskotin M., Tretyakov I., Yagodkin D., Zhukov S., Taniguchi T. (2018). Resonant terahertz detection using graphene plasmons. Nat. Commun..

[113] Lu Y., Feng S., Wu Z., Gao Y., Yang J., Zhang Y., Hao Z., Li J., Li E., Chen H. (2018). Broadband surface plasmon resonance enhanced self-powered graphene/GaAs photodetector with ultrahigh detectivity. Nano Energy.

[114] Wang G., Li L., Fan W., Wang R., Zhou S., Lü J.-T., Gan L., Zhai T. (2018). Interlayer Coupling Induced Infrared Response in WS2/MoS2 Heterostructures Enhanced by Surface Plasmon Resonance. Adv. Funct. Mater..

[115] Ahmed A.A., Hashim M.R., Abdalrheem R., Rashid M. (2019). High-performance multicolor metal-semiconductor-metal Si photodetector enhanced by nanostructured NiO thin film. J. Alloys Compd..

[116] Simola E.T., De Iacovo A., Frigerio J., Ballabio A., Fabbri A., Isella G., Colace L. (2019). Voltage-tunable dual-band Ge/Si photodetector operating in VIS and NIR spectral range. Opt. Express.

[117] Zheng J.J., Chong H.N., Wang L., Chen S.L., Yang W.Y., Wei G.D., Gao F.M. (2020). A robust SiC nanoarray blue-light photodetector. J. Mater. Chem. C.

[118] Guo D.Y., Su Y.L., Shi H.Z., Li P.G., Zhao N., Ye J.H., Wang S.L., Liu A.P., Chen Z.W., Li C.R. (2018). Self-Powered Ultraviolet Photodetector with Superhigh Photoresponsivity (3.05 A/W) Based on the GaN/Sn:Ga2O3 pn Junction. ACS Nano.

[119] Tao L., Chen Z., Li Z., Wang J., Xu X., Xu J.B. (2020). Enhancing light-matter interaction in 2D materials by optical micro/nano architectures for high-performance optoelectronic devices. InfoMat.

[120] Chen X., Shehzad K., Gao L., Long M., Guo H., Qin S., Wang X., Wang F., Shi Y., Hu W. (2020). Graphene Hybrid Structures for Integrated and Flexible Optoelectronics. Adv. Mater..

[121] Guo Q., Pospischil A., Bhuiyan M., Jiang H., Tian H., Farmer D., Deng B., Li C., Han S.J., Wang H. (2016). Black Phosphorus Mid-Infrared Photodetectors with High Gain. Nano Lett..

[122] Nguyen D.A., Park D.Y., Lee J., Duong N.T., Park C., Nguyen D.H., Le T.S., Suh D., Yang H., Jeong M.S. (2021). Patterning of type-II Dirac semimetal PtTe2 for optimized interface of tellurene optoelectronic device. Nano Energy.

[123] Shen P.C., Su C., Lin Y.X., Chou A.S., Cheng C.C., Park J.H., Chiu M.H., Lu A.Y., Tang H.L., Tavakoli M.M. (2021). Ultralow contact resistance between semimetal and monolayer semiconductors. Nature.

[124] Zhang X., Liu B., Gao L., Yu H., Liu X., Du J., Xiao J., Liu Y., Gu L., Liao Q. (2021). Near-ideal van der Waals rectifiers based on all-two-dimensional Schottky junctions. Nat. Commun..

[125] Song S., Sim Y., Kim S.-Y., Kim J.H., Oh I., Na W., Lee D.H., Wang J., Yan S., Liu Y. (2020). Wafer-scale production of patterned transition metal ditelluride layers for two-dimensional metal–semiconductor contacts at the Schottky–Mott limit. Nat. Electron..

[126] Yin Z.Y., Li H., Li H., Jiang L., Shi Y.M., Sun Y.H., Lu G., Zhang Q., Chen X.D., Zhang H. (2012). Single-Layer MoS2 Phototransistors. ACS Nano.

[127] Furchi M.M., Polyushkin D.K., Pospischil A., Mueller T. (2014). Mechanisms of photoconductivity in atomically thin MoS2. Nano Lett..

[128] Buscema M., Barkelid M., Zwiller V., van der Zant H.S., Steele G.A., Castellanos-Gomez A. (2013). Large and tunable photothermoelectric effect in single-layer MoS2. Nano Lett..

[129] Choi W., Cho M.Y., Konar A., Lee J.H., Cha G.B., Hong S.C., Kim S., Kim J., Jena D., Joo J. (2012). High-detectivity multilayer MoS(2) phototransistors with spectral response from ultraviolet to infrared. Adv. Mater..

[130] Xia F., Mueller T., Lin Y.M., Valdes-Garcia A., Avouris P. (2009). Ultrafast graphene photodetector. Nat. Nanotechnol..

[131] Wang Q., Zhou C., Chai Y. (2020). Breaking symmetry in device design for self-driven 2D material based photodetectors. Nanoscale.

[132] Huang Z., Jiang Y., Han Q., Yang M., Han J., Wang F., Luo M., Li Q., Zhu H., Liu X. (2020). High responsivity and fast UV-vis-short-wavelength IR photodetector based on Cd3As2/MoS2 heterojunction. Nanotechnology.

[133] Lai J., Liu X., Ma J., Wang Q., Zhang K., Ren X., Liu Y., Gu Q., Zhuo X., Lu W. (2018). Anisotropic Broadband Photoresponse of Layered Type-II Weyl Semimetal MoTe2. Adv. Mater..

[134] Farooq Khan M., Arslan Shehzad M., Zahir Iqbal M., Waqas Iqbal M., Nazir G., Seo Y., Eom J. (2017). A facile route to a high-quality graphene/MoS2 vertical field-effect transistor with gate-modulated photocurrent response. J. Mater. Chem. C.

[135] Riazimehr S., Kataria S., Gonzalez-Medina J.M., Wagner S., Shaygan M., Suckow S., Ruiz F.G., Engstrom O., Godoy A., Lemme M.C. (2019). High Responsivity and Quantum Efficiency of Graphene/Silicon Photodiodes Achieved by Interdigitating Schottky and Gated Regions. Acs Photonics.

[136] Giannazzo F., Schilirò E., Lo Nigro R., Prystawko P., Cordier Y., Roccaforte F., Leszczynski M. (2020). Integration of 2D Materials with Nitrides for Novel Electronic and Optoelectronic Applications. Nitride Semiconductor Technology.

[137] Ren H., Chen J.D., Li Y.Q., Tang J.X. (2021). Recent Progress in Organic Photodetectors and their Applications. Adv. Sci..

[138] Qiao H., Huang Z., Ren X., Liu S., Zhang Y., Qi X., Zhang H. (2019). Self-Powered Photodetectors Based on 2D Materials. Adv. Opt. Mater..

[139] Tan C., Yin S., Chen J., Lu Y., Wei W., Du H., Liu K., Wang F., Zhai T., Li L. (2021). Broken-Gap PtS2/WSe2 van der Waals Heterojunction with Ultrahigh Reverse Rectification and Fast Photoresponse. ACS Nano.

[140] Tao J.J., Jiang J., Zhao S.N., Zhang Y., Li X.X., Fang X., Wang P., Hu W., Lee Y.H., Lu H.L. (2021). Fabrication of 1D Te/2D ReS2 Mixed-Dimensional van der Waals p-n Heterojunction for High-Performance Phototransistor. ACS Nano.

[141] Zou Z., Liang J., Zhang X., Ma C., Xu P., Yang X., Zeng Z., Sun X., Zhu C., Liang D. (2021). Liquid-Metal-Assisted Growth of Vertical GaSe/MoS2 p-n Heterojunctions for Sensitive Self-Driven Photodetectors. ACS Nano.

[142] Luo P., Wang F.K., Qu J.Y., Liu K.L., Hu X.Z., Liu K.W., Zhai T.Y. (2021). Self-Driven WSe2/Bi2O2Se Van der Waals Heterostructure Photodetectors with High Light On/Off Ratio and Fast Response. Adv. Funct. Mater..

[143] Gong F., Fang H., Wang P., Su M., Li Q., Ho J.C., Chen X., Lu W., Liao L., Wang J. (2017). Visible to near-infrared photodetectors based on MoS2vertical Schottky junctions. Nanotechnology.

[144] Kufer D., Nikitskiy I., Lasanta T., Navickaite G., Koppens F.H., Konstantatos G. (2015). Hybrid 2D-0D MoS2 -PbS quantum dot photodetectors. Adv. Mater..

[145] Huo N., Gupta S., Konstantatos G. (2017). MoS2 -HgTe Quantum Dot Hybrid Photodetectors beyond 2 microm. Adv. Mater..

[146] Karnatak P., Paul T., Islam S., Ghosh A. (2017). 1/f noise in van der Waals materials and hybrids. Adv. Phys.-X.

[147] Balandin A.A. (2013). Low-frequency 1/f noise in graphene devices. Nat. Nanotechnol..

[148] Furchi M., Urich A., Pospischil A., Lilley G., Unterrainer K., Detz H., Klang P., Andrews A.M., Schrenk W., Strasser G. (2012). Microcavity-integrated graphene photodetector. Nano Lett..

[149] Engel M., Steiner M., Lombardo A., Ferrari A.C., Lohneysen H.V., Avouris P., Krupke R. (2012). Light-matter interaction in a microcavity-controlled graphene transistor. Nat. Commun..

[150] Wang X.M., Cheng Z.Z., Xu K., Tsang H.K., Xu J.B. (2013). High-responsivity graphene/silicon-heterostructure waveguide photodetectors. Nat. Photonics.

[151] Gan X., Shiue R.-J., Gao Y., Meric I., Heinz T.F., Shepard K., Hone J., Assefa S., Englund D. (2013). Chip-integrated ultrafast graphene photodetector with high responsivity. Nat. Photonics.

[152] Goykhman I., Sassi U., Desiatov B., Mazurski N., Milana S., de Fazio D., Eiden A., Khurgin J., Shappir J., Levy U. (2016). On-Chip Integrated, Silicon-Graphene Plasmonic Schottky Photodetector with High Responsivity and Avalanche Photogain. Nano Lett..

[153] Pospischil A., Humer M., Furchi M.M., Bachmann D., Guider R., Fromherz T., Mueller T. (2013). CMOS-compatible graphene photodetector covering all optical communication bands. Nat. Photonics.

[154] Wu Z., Zhang T., Chen Y., Zhang Y., Yu S. (2019). Integrating Graphene/MoS2 Heterostructure with SiNx Waveguide for Visible Light Detection at 532 nm Wavelength. Phys. Status Solidi-Rapid Res. Lett..

[155] Zhan T.R., Zhao F.Y., Hu X.H., Liu X.H., Zi J. (2012). Band structure of plasmons and optical absorption enhancement in graphene on subwavelength dielectric gratings at infrared frequencies. Phys. Rev. B.

[156] Guo J., Li S., He Z., Li Y., Lei Z., Liu Y., Huang W., Gong T., Ai Q., Mao L. (2019). Near-infrared photodetector based on few-layer MoS2 with sensitivity enhanced by localized surface plasmon resonance. Appl. Surf. Sci..

[157] Lan H.Y., Hsieh Y.H., Chiao Z.Y., Jariwala D., Shih M.H., Yen T.J., Hess O., Lu Y.J. (2021). Gate-Tunable Plasmon-Enhanced Photodetection in a Monolayer MoS2 Phototransistor with Ultrahigh Photoresponsivity. Nano Lett..

[158] Schedin F., Lidorikis E., Lombardo A., Kravets V.G., Geim A.K., Grigorenko A.N., Novoselov K.S., Ferrari A.C. (2010). Surface-Enhanced Raman Spectroscopy of Graphene. ACS Nano.

[159] Salamin Y., Ma P., Baeuerle B., Emboras A., Fedoryshyn Y., Heni W., Cheng B., Josten A., Leuthold J. (2018). 100 GHz Plasmonic Photodetector. Acs Photonics.

[160] Miao J., Hu W., Jing Y., Luo W., Liao L., Pan A., Wu S., Cheng J., Chen X., Lu W. (2015). Surface Plasmon-Enhanced Photodetection in Few Layer MoS2 Phototransistors with Au Nanostructure Arrays. Small.

[161] Kim M., Kang P., Leem J., Nam S. (2017). A stretchable crumpled graphene photodetector with plasmonically enhanced photoresponsivity. Nanoscale.

[162] Huang J.-A., Luo L.-B. (2018). Low-Dimensional Plasmonic Photodetectors: Recent Progress and Future Opportunities. Adv. Opt. Mater..

[163] Koppens F.H., Mueller T., Avouris P., Ferrari A.C., Vitiello M.S., Polini M. (2014). Photodetectors based on graphene, other two-dimensional materials and hybrid systems. Nat. Nanotechnol..

[164] Lou Z., Liang Z., Shen G. (2016). Photodetectors based on two dimensional materials. J. Semicond..

[165] Wang P., Liu S., Luo W., Fang H., Gong F., Guo N., Chen Z.G., Zou J., Huang Y., Zhou X. (2017). Arrayed Van Der Waals Broadband Detectors for Dual-Band Detection. Adv. Mater..

[166] Goossens S., Navickaite G., Monasterio C., Gupta S., Piqueras J.J., Pérez R., Burwell G., Nikitskiy I., Lasanta T., Galán T. (2017). Broadband image sensor array based on graphene-CMOS integration. Nat. Photonics.

[167] Das S., Pandey D., Thomas J., Roy T. (2019). The Role of Graphene and Other 2D Materials in Solar Photovoltaics. Adv. Mater..

[168] Tsai M.L., Li M.Y., Retamal J.R.D., Lam K.T., Lin Y.C., Suenaga K., Chen L.J., Liang G., Li L.J., He J.H. (2017). Single Atomically Sharp Lateral Monolayer p-n Heterojunction Solar Cells with Extraordinarily High Power Conversion Efficiency. Adv. Mater..

[169] Wong J., Jariwala D., Tagliabue G., Tat K., Davoyan A.R., Sherrott M.C., Atwater H.A. (2017). High Photovoltaic Quantum Efficiency in Ultrathin van der Waals Heterostructures. ACS Nano.

[170] Yao J., Yang G. (2020). 2D material broadband photodetectors. Nanoscale.

[171] Stewart J.W., Vella J.H., Li W., Fan S., Mikkelsen M.H. (2020). Ultrafast pyroelectric photodetection with on-chip spectral filters. Nat. Mater..

[172] Chen Y., Wang Y., Wang Z., Gu Y., Ye Y., Chai X., Ye J., Chen Y., Xie R., Zhou Y. (2021). Unipolar barrier photodetectors based on van der Waals heterostructures. Nature Electronics.

[173] Wang D.K., Chen X., Fang X., Tang J.L., Lin F.Y., Wang X.W., Liu G.L., Liao L., Ho J.C., Wei Z.P. (2021). Photoresponse improvement of mixed-dimensional 1D-2D GaAs photodetectors by incorporating constructive interface states. Nanoscale.

[174] Geng H., Yuan D., Yang Z., Tang Z., Zhang X., Yang K., Su Y. (2019). Graphene van der Waals heterostructures for high-performance photodetectors. J. Mater. Chem. C.

[175] Jang H., Seok Y., Choi Y., Cho S.H., Watanabe K., Taniguchi T., Lee K. (2021). High-Performance Near-Infrared Photodetectors Based on Surface-Doped InSe. Adv. Funct. Mater..

[176] Wei T., Wang X., Yang Q., He Z., Yu P., Xie Z., Chen H., Li S., Wu S. (2021). Mid-Infrared Photodetection of Type-II Dirac Semimetal 1T-PtTe2 Grown by Molecular Beam Epitaxy. ACS Appl. Mater. Interfaces.

[177] Konstantatos G., Sargent E.H. (2010). Nanostructured materials for photon detection. Nat. Nanotechnol..

[178] Wang A.Q., Ye X.G., Yu D.P., Liao Z.M. (2020). Topological Semimetal Nanostructures: From Properties to Topotronics. ACS Nano.

